# Novel insights into the intraepithelial spread of extrahepatic cholangiocarcinoma: clinicopathological study of 382 cases on extrahepatic cholangiocarcinoma

**DOI:** 10.3389/fonc.2023.1216097

**Published:** 2023-08-17

**Authors:** Daisuke Nagashima, Minoru Esaki, Satoshi Nara, Daisuke Ban, Takeshi Takamoto, Takahiro Mizui, Kazuaki Shimada, Nobuyoshi Hiraoka

**Affiliations:** ^1^ Division of Molecular Pathology, National Cancer Center Research Institute, Tokyo, Japan; ^2^ Department of Hepatobiliary and Pancreatic Surgery, National Cancer Center Hospital, Tokyo, Japan; ^3^ Department of Molecular Oncology, Jikei University Graduate School of Medicine, Tokyo, Japan; ^4^ Division of Innovative Pathology and Laboratory Medicine, National Cancer Center Exploratory Oncology Research & Clinical Trial Center (EPOC), Tokyo, Japan

**Keywords:** intraepithelial spread, extrahepatic cholangiocarcinoma, biliary tract cancer, tumor location, patient outcome

## Abstract

**Background:**

Extrahepatic cholangiocarcinoma (eCCA) is a rare and aggressive disease and consisted of conventional eCCA and intraductal papillary neoplasm of the bile duct (IPNB). Intraepithelial spread (IES) of cancer cells beyond the invasive area is often observed in IPNBs; however, the prevalence of IES remains to be examined in conventional eCCAs. Here, we evaluated the clinicopathological features of eCCAs according to tumor location, with a focus on the presence of IES. The IES extension was also compared among biliary tract cancers (BTCs).

**Methods:**

We examined the prevalence and clinicopathological significance of IES in eCCAs (n=382) and the IES extension of BTCs, including gallbladder (n=172), cystic duct (n=20), and ampullary cancers (n=102).

**Results:**

Among the invasive eCCAs, IPNB had a higher rate of IES (89.2%) than conventional eCCAs (57.0%). Among conventional eCCAs, distal eCCAs (75.4%) had a significantly higher prevalence of IES than perihilar eCCAs (41.3%). The presence of IES was associated with a significantly higher survival rate in patients with distal eCCAs (*P*=0.030). Extension of the IES into the cystic duct (CyD) in distal eCCAs that cancer cells reached the junction of the CyD was a favorable prognostic factor (*P*<0.001). The association of survival with IES, either on the extrahepatic bile duct or on the CyD, differed depending on the tumor location and type of eCCA. The extension properties of IES were also dependent on different types of tumors among BTCs; usually, the IES incidence became higher than 50% in the tissues that the tumor developed, whereas IES extension to other tissues decreased the incidence.

**Conclusion:**

Thus, eCCAs have different clinicopathological characteristics depending on the tumor location and type.

## Introduction

The biliary tract comprises the intrahepatic bile duct (IHBD), extrahepatic bile duct (EHBD), cystic duct (CyD), gallbladder, and ampulla of Vater. Biliary tract cancers (BTCs) are rare and aggressive, and because of limited treatment options, they are associated with poor outcomes (1, 2). Extrahepatic cholangiocarcinoma (eCCA) accounts for approximately one-third of BTCs (3). The incidence of eCCA varies geographically, with a high incidence in east Asia, although it has increased worldwide (4, 5). Owing to the differences of clinicopathological characteristics of eCCAs dependent on anatomical location, eCCAs are currently categorized as perihilar and distal eCCAs to be evaluated in different tumor-node-metastasis (TNM) classifications (6). The accumulated findings suggest that the differences of eCCAs characteristics may be based on not only simply location differences but also biological properties of cancer cells raised in different anatomical locations (7–10). Thus, further clinicopathological characterization and exploration of molecular alterations in eCCA are needed.

Intraepithelial spread (IES) of cancer cells beyond the invasive area is found in several cancers, including BTCs (11–16). Extensive IES may represent a less aggressive behavior of the tumor and is associated with better patient outcome in eCCA (11, 13, 15) and pancreatic ductal adenocarcinoma (PDAC) (12, 16). Recent reports also indicated that the presence of IES without invasive cancer cells in the bile duct margin is not an unfavorable factor (14, 17–19). The previous studies have characterized eCCAs with extensive IES as unique eCCAs that show macroscopic papillary type and histological papillary adenocarcinoma with a high incidence and long-term prognosis. These features of tumors with extensive IES are very similar to those of intraductal papillary neoplasms of the bile duct (IPNBs) and its derived invasive cancers, entities that first appeared in the 2010 World Health Organization (WHO) classification after published reports. IPNBs are grossly visible premalignant neoplasms with intraductal papillary or villous growth of epithelial neoplastic cells, and intrahepatic IPNBs show better outcome compared to extrahepatic IPNBs (20). Since previous IES studies analyzed eCCAs without dividing IPNBs from the conventional eCCAs (11, 13, 15), it remains to be investigated if reported characteristics of IES are also relevant to conventional eCCAs. In addition, the extensive properties of the IES have not been characterized, especially the extension of the IES beyond the borders among different tissues. The EHBD connects continuously to different tissues, such as the CyD and ampullary ducts, and through them to the gallbladder and duodenum.

In this study, we investigated the clinicopathological features of eCCAs (n=382) in terms of tumor location, with a focus on the presence of IES. We also compared the incidence and extension properties of IES among BTCs, including eCCAs, gallbladder cancers (GBCs, n=172), cystic duct cancers (CyDCs, n=20), and ampullary cancers (AVCs, n=102).

## Materials and methods

### Patients of eCCAs

We retrospectively evaluated 382 eCCA patients who underwent surgical resection at the National Cancer Center Hospital between January 2002 and March 2022. All the patients included in this study underwent macroscopic curative resection of conventional eCCAs or IPNBs that developed during EHBD. We excluded patients who had received any therapy before surgery and those with inadequate IES data. In addition, cases of hospital death after surgical resection, cases with unknown causes of death, or early death not due to eCCAs within 12 months after surgical resection were excluded. Finally, 305 patients were included in this study. For survival analyses, cases of carcinoma *in situ* of conventional eCCA were excluded. **Supplementary Figure 1** describes the details of patient selection.

Surgical procedures were performed based on the location of the primary tumor. Among 305 patients, 140 (45.9%) underwent hepatectomy with extrahepatic bile duct resection, 124 (40.7%) underwent pancreaticoduodenectomy, 17 (5.6%) underwent combined hepatectomy and pancreaticoduodenectomy, and 24 (7.9%) underwent extrahepatic bile duct resection. Para-aortic lymph node sampling was performed when lymph node metastasis was suspected. In our hospital, adjuvant therapy is not routinely performed after surgery, although adjuvant S-1 therapy has become a standard of care according to the results of the JCOG1202 study since October 2021 (21). Only four patients underwent adjuvant chemotherapy with S-1 during the study period. Clinical and radiological follow-ups were scheduled on a 3-month basis for a few years after resection. The median follow-up period was 41.0 months for all 305 patients. Recurrence was confirmed by radiological examination and elevation of tumor markers. The date of recurrence was defined as the date on which clinicians confirmed recurrence in medical records. The census date was December 31, 2022. According to previous studies, early recurrence is defined as any recurrence within 12 months after surgery (22, 23).

### Patients with GBCs, CyDCs, or AVCs

To assess the incidence and extent of IES in BTCs, 102 cases of AVC, 174 cases of GBC, and 20 cases of CyDC were included, all of which were surgically resected at the National Cancer Center Hospital between January 2002 and March 2022. **Supplementary Table 1** shows the demographics of the patients with GBC, AVC, and CyDC. AVCs raised in the common, bile, or pancreatic ducts of the ampulla of Vater were selected for this assessment.

### Pathological examination

All of the BTCs were examined pathologically and classified according to the WHO classification (2, 24, 25) and the International Union against Cancer (UICC) TNM classification 8th edition (6). We had some modification about tumor location as mentioned later. Macroscopic types of eCCA and the following histopathological variables were evaluated following the Japanese Society of Biliary Surgery (JSBS) classification (26): lymphatic, venous, and perineural invasions that were classified into negative (–), slightly positive (1+), moderately positive (2+), and markedly positive (3+) based on their event frequencies. For the survival and correlation analyses, “high” and “low” grades were determined based on these values; high grade to be combined with 2+ and 3+ and low grade to be - and 1+. According to the JSBS classification (27), the right and left hepatic ducts and their confluence were defined as the (peri)hilar duct (Bph), common hepatic duct, and common bile duct, and were divided into three portions as follows: superior (Bs), middle (Bm), and inferior (Bi) portions of the EHBD. Bs and Bm were defined as the respective portions in the upper and lower halves of the bile duct length from the confluence of the right and left hepatic ducts to the upper margin of the pancreas, and Bi was defined as the portion from the upper margin of the pancreas to the ampulla of Vater. In some analyses, we combined Bph and Bs eCCAs as perihilar eCCAs, and Bm and Bi eCCAs as distal eCCAs. IHBD was defined as the hepatic side of the bile duct from the third branch (e.g., segmental ducts 5 and 8) of the hepatic duct in this study. All patients with stage IV disease were diagnosed on the basis of para-aortic lymph node involvement. Surgically resected specimens were fixed in 10% formalin and cut into serial slices 5 mm thick. All sections were stained with hematoxylin and eosin for pathological examination. IES was defined as the intraepithelial spread of cancer cells beyond the invasive area. IES contained lesions corresponded to biliary intraepithelial neoplasia, high grade (BilIN-3), intraductal papillary neoplasm of the bile duct (IPNB), intracholecystic papillary neoplasm (ICPN), intraampullary papillary tubular neoplasm (IAPN), and cancerous duct. IES was diagnosed only when the cancer cells extended along the biliary tract. IES was not applied when cancer cells had stromal invasion beyond the biliary tract structure without extension along the biliary tract and the cancer cells re-entered the biliary tract mucosal epithelial layer. We defined that “intraepithelial extension of cancer cells on duodenum” was present when intraepithelial extension of cancer cells on ampullary common duct continued to extend to the duodenum epithelial layer. The length of the IES was described using a 5 mm scale in general.

### Statistical analysis

Statistical analyses were performed using JMP version 12.2 (SAS Institute, Cary, NC, USA) and the StatView-J software version 5.0 (Abacus Concepts, Berkeley, CA, USA). Continuous data were expressed as median (range) and compared using the Mann–Whitney U test. Categorical variables were compared between groups using Pearson’s chi-square test or Fisher’s exact test, as appropriate. Relapse-free survival (RFS) was defined as the interval between the date of surgery and time of recurrence. Overall survival (OS) was calculated based on the time from surgery to death from any cause or last follow-up. Survival data were estimated using the Kaplan–Meier method and examined using the log-rank test. Factors found to be significant in the univariate analysis were subjected to multivariate analysis using the Cox proportional hazards model. Differences at *P* < 0.05 were considered statistically significant.

## Results

### Clinicopathological characteristics of eCCAs

Details of the surgical and clinicopathological features are presented in **Table 1**. Among 284 patients with invasive eCCAs, those with Bph eCCAs were significantly younger than those with other conventional eCCAs. The female ratio in Bph eCCAs was significantly higher than that in Bs and Bi eCCAs, and similar tendencies were found in Bm eCCAs and invasive IPNBs. The total tumor sizes of Bph eCCAs were significantly smaller than those of Bs and Bm eCCAs, whereas the sizes of the tumor area with stromal invasion of cancer cells (invasive tumor sizes) of Bi eCCAs and invasive IPNBs were significantly smaller than those of the other conventional eCCAs.

**Table 1A:** Clinicopathological variables of invasive extrahepatic cholangiocarcinomas (n = 284).

	Bph eCCA n= 96	Bs eCCA n= 42	Bm eCCA n= 59	Bi eCCA n= 59	IPNB n= 28
Age, year [range]	65 [19-83]	70 [39-87]	68 [44-82]	71 [41-83]	74.5 [33-82]
Female/Male	29/67	5/37	12/47	10/49	4/24
Macroscopic type					
nodular-infiltrating	78 (81.3)	33 (78.6)	47 (79.7)	40 (67.8)	2 (7.1)
nodular-expanding	1 (1.0)	0 (0)	1 (1.7)	3 (5.1)	1 (3.6)
papillary-infiltrating	3 (3.1)	4 (9.5)	4 (6.8)	8 (13.6)	17 (60.7)
papillary-expanding	0 (0)	1 (2.4)	1 (1.7)	0 (0)	8 (28.6)
flat-infiltrating	13 (13.5)	4 (9.5)	6 (10.2)	8 (13.6)	0 (0)
flat-expanding	1 (1.0)	0 (0)	0 (0)	0 (0)	0 (0)
Total tumor size, mm [range]	40 [15-150]	50 [20-90]	55 [15-120]	50 [20-100]	57.5 [15-120]
Invasive tumor size, mm [range]	35 [15-120]	35 [20-90]	40 [15-70]	30 [15-70]	22.5 [5-50]
Histology (histological grade)					
Tub1 (G1)	19 (19.8)	9 (21.4)	10 (17.0)	10 (17.0)	3 (10.7)
Tub2 (G2)	71 (74.0)	23 (54.8)	37 (62.7)	30 (50.8)	6 (21.4)
Por (G3)	4 (4.2)	8 (19.1)	10 (17.0)	15 (25.4)	1 (3.6)
Pap (G1)	0 (0)	1 (2.4)	2 (3.4)	2 (3.4)	18 (64.3)
AS (G3)	2 (2.1)	1 (2.4)	0 (0)	2 (3.4)	0 (0)
Depth of invasion					
fm	0 (0)	1 (2.4)	1 (1.7)	0 (0)	7 (25.0)
ss	88 (91.7)	38 (90.5)	58 (98.3)	59 (100)	20 (71.4)
se	7 (7.3)	2 (4.8)	0 (0)	0 (0)	1 (3.6)
si	1 (1.0)	1 (2.4)	0 (0)	0 (0)	0 (0)
Portal vein invasion					
presence	39 (40.6)	2 (4.8)	5 (8.5)	1 (1.7)	1 (3.6)
absence	57 (59.4)	40 (95.2)	54 (91.5)	58 (98.3)	27 (96.4)
Artery invasion					
presence	12 (12.5)	7 (16.7)	0 (0)	0 (0)	0 (0)
absence	84 (87.5)	35 (83.3)	59 (100)	59 (100)	28 (100)
Lymphatic invasion					
high	46 (47.9)	15 (35.7)	31 (52.5)	29 (49.2)	8 (28.6)
low	50 (52.1)	27 (64.3)	28 (47.5)	30 (50.8)	20 (71.4)
Venous invasion					
high	64 (66.7)	20 (47.6)	25 (42.4)	27 (45.8)	6 (21.4)
low	32 (33.3)	22 (52.4)	34 (57.6)	32 (54.2)	22 (78.6)
Perineural invasion					
high	72 (75.0)	38 (90.5)	52 (88.1)	44 (74.6)	8 (28.6)
low	24 (25.0)	4 (9.5)	7 (11.9)	15 (25.4)	20 (71.4)
Surgical procedure					
EBDR	2 (2.1)	7 (16.7)	7 (11.9)	1 (1.7)	6 (21.4)
PD	0 (0)	6 (14.3)	41 (69.5)	57 (96.6)	17 (60.7)
Hepatectomy	87 (90.6)	28 (66.7)	5 (8.5)	0 (0)	4 (14.3)
HPD	7 (7.3)	1 (2.4)	6 (10.2)	1 (1.7)	1 (3.6)
Major vessel resection					
PVR	15 (15.6)	2 (4.8)	10 (17.0)	2 (3.4)	0 (0)
HAR	5 (5.4)	3 (7.1)	1 (1.7)	1 (1.7)	0 (0)
PVR+HAR	7 (7.5)	0 (0)	1 (1.7)	0 (0)	0 (0)
Bile duct margin status					
presence with invasive cancer	24 (25.0)	12 (28.6)	8 (13.6)	2 (3.4)	0 (0)
presence with non-invasive cancer	14 (14.6)	11 (26.2)	18 (30.5)	11 (18.6)	8 (28.6)
absence	58 (60.4)	19 (45.2)	33 (55.9)	46 (78.0)	20 (71.4)
Residual tumor status					
microscopic residual tumor	40 (41.7)	24 (57.1)	32 (54.2)	16 (27.1)	10 (35.7)
no residual tumor	56 (58.3)	18 (42.9)	27 (45.8)	43 (72.9)	18 (64.3)
Recurrence					
presence	71 (74.0)	27 (64.3)	41 (69.5)	30 (50.8)	15 (53.6)
absence	25 (26.0)	15 (35.7)	18 (30.5)	29 (49.2)	13 (46.4)
Early recurrence					
presence	29 (30.2)	10 (23.8)	22 (37.3)	15 (25.4)	5 (17.9)
absence	67 (69.8)	32 (76.2)	37 (62.7)	44 (74.6)	23 (82.1)

Values given are the number of patients (percentage) unless otherwise indicated.

AS, adenosquamous carcinoma; Bi, inferior portion of EHBD; Bm, middle portion of EHBD; Bph, (peri)hilar bile duct; Bs, superior portion of EHBD; eCCA, extrahepatic cholangiocarcinoma; EHBD, extrahepatic bile duct; EBDR, extrahepatic bile duct resection; fm, fibromusclar layer; HAR, hepatic artery resection; HPD, hepatopancreatoduodenectomy; IPNB, intraductal papillary neoplasm of the bile duct; Pap, papillary adenocarcinoma; Por, poorly differentiated adenocarcinoma; PD, pancreaticoduodenectomy; PVR, Portal vein resection; se, exposed on serosal surface; si, infiltration beyond the serosa to other tissues; ss, subserosal tissue; Tub1, well differentiated tubular adenocarcinoma; Tub2, moderately differentiated tubular adenocarcinoma.

**Table 1B:** Clinicopathological variables of invasive extrahepatic cholangiocarcinomas (n = 284).

	Bph eCCA n= 96	Bs eCCA n= 42	IPNB-perihilar n= 8		Bm eCCA n= 59	Bi eCCA n= 59	IPNB-distal n= 20
TMN classification							
T category							
T1	0 (0)	11 (26.2)	5 (62.5)	T1	19 (32.2)	7 (11.9)	11 (55.0)
T2a	19 (19.8)	25 (59.5)	2 (25.0)	T2	30 (50.8)	42 (71.2)	6 (30.0)
T2b	36 (37.5)	2 (4.8)	1 (12.5)	T3	10 (17.0)	10 (17.0)	3 (15.0)
T3	31 (32.3)	4 (9.5)	0 (0)	T4	0 (0)	0 (0)	0 (0)
T4	10 (10.4)	0 (0)	0 (0)				
N category							
N0	49 (51.0)	17 (40.5)	7 (87.5)	N0	26 (44.1)	35 (59.3)	13 (65.0)
N1	33 (34.4)	19 (45.2)	1 (12.5)	N1	24 (40.7)	15 (25.4)	2 (10.0)
N2	14 (14.6)	6 (14.3)	0 (0)	N2	9 (15.3)	9 (15.3)	5 (25.0)
M category							
M0	91 (94.8)	38 (90.5)	8 (100)	M0	57 (96.6)	55 (93.2)	18 (90.0)
M1	5 (5.2)	4 (9.5)	0 (0)	M1	2 (3.4)	4 (6.8)	2 (10.0)
Stage							
I	0 (0)	6 (14.3)	5 (62.5)	I	9 (15.3)	7 (11.9)	9 (45.0)
II	35 (36.5)	9 (21.4)	2 (25.0)	IIA	22 (37.3)	24 (40.7)	5 (25.0)
IIIA	10 (10.4)	2 (4.8)	0 (0)	IIB	17 (28.8)	18 (30.5)	1 (5.0)
IIIB	3 (3.1)	0 (0)	0 (0)	IIIA	9 (15.3)	6 (10.2)	3 (15.0)
IIIC	31 (32.3)	17 (40.5)	1 (12.5)	IIIB	0 (0)	0 (0)	0 (0)
IVA	12 (12.5)	4 (9.5)	0 (0)	IV	2 (3.4)	4 (6.8)	2 (10.0)
IVB	5 (5.2)	4 (9.5)	0 (0)				

Values given are the number of patients (percentage) unless otherwise indicated.

IPNB was not classified by tumor location due to small number of cases.

Approximately 80% of conventional invasive eCCAs belong to the nodular-infiltrating macroscopic type. In contrast, more than 85% of invasive IPNB cases were papillary types. The incidence of poorly differentiated adenocarcinomas in Bph eCCAs was significantly lower than that in other conventional eCCAs. Invasive IPNBs predominantly include papillary adenocarcinomas. The invasion depth tended to be lower in invasive IPNBs than in conventional eCCAs. The frequencies of portal vein or artery invasion of cancer cells were much higher in Bph and Bs eCCAs, as the portal vein or artery exists nearer to the EHBD in these regions compared to other sites. The lymphatic, vascular, and perineural invasion of tumor cells tended to be lower in invasive IPNBs than in conventional eCCAs. Venous invasion was significantly higher in Bph eCCAs than in other conventional eCCAs and invasive IPNBs. Perineural invasion was significantly lower in Bi eCCAs than in Bs and Bm eCCAs. The rates of residual tumor-free tumors (R0) were significantly higher in Bi eCCAs than in other conventional eCCAs. The rates of positive bile duct margins with invasive cancers and positive residual tumor status with invasive cancers were significantly higher in Bph and Bs eCCAs than in Bi eCCA and invasive IPNBs.

Patients with noninvasive IPNBs had significantly better survival than those with invasive eCCAs, including invasive IPNBs (**Supplementary Figure 2**). The survival outcomes of invasive IPNBs were significantly better than those of Bm or Bph eCCAs, but not significantly different from those of Bs or Bi eCCAs. The survival curve of Bi eCCAs was similar to that of invasive IPNBs for both RFS and OS, and the 5-year and 10-year survival rates were similar. The survival outcomes of patients with Bi eCCAs were significantly better than those of patients with Bph and Bm eCCAs in terms of both RFS and OS.

### Clinicopathological characteristics of invasive eCCAs with IES along EHBD

**Figure 1:**
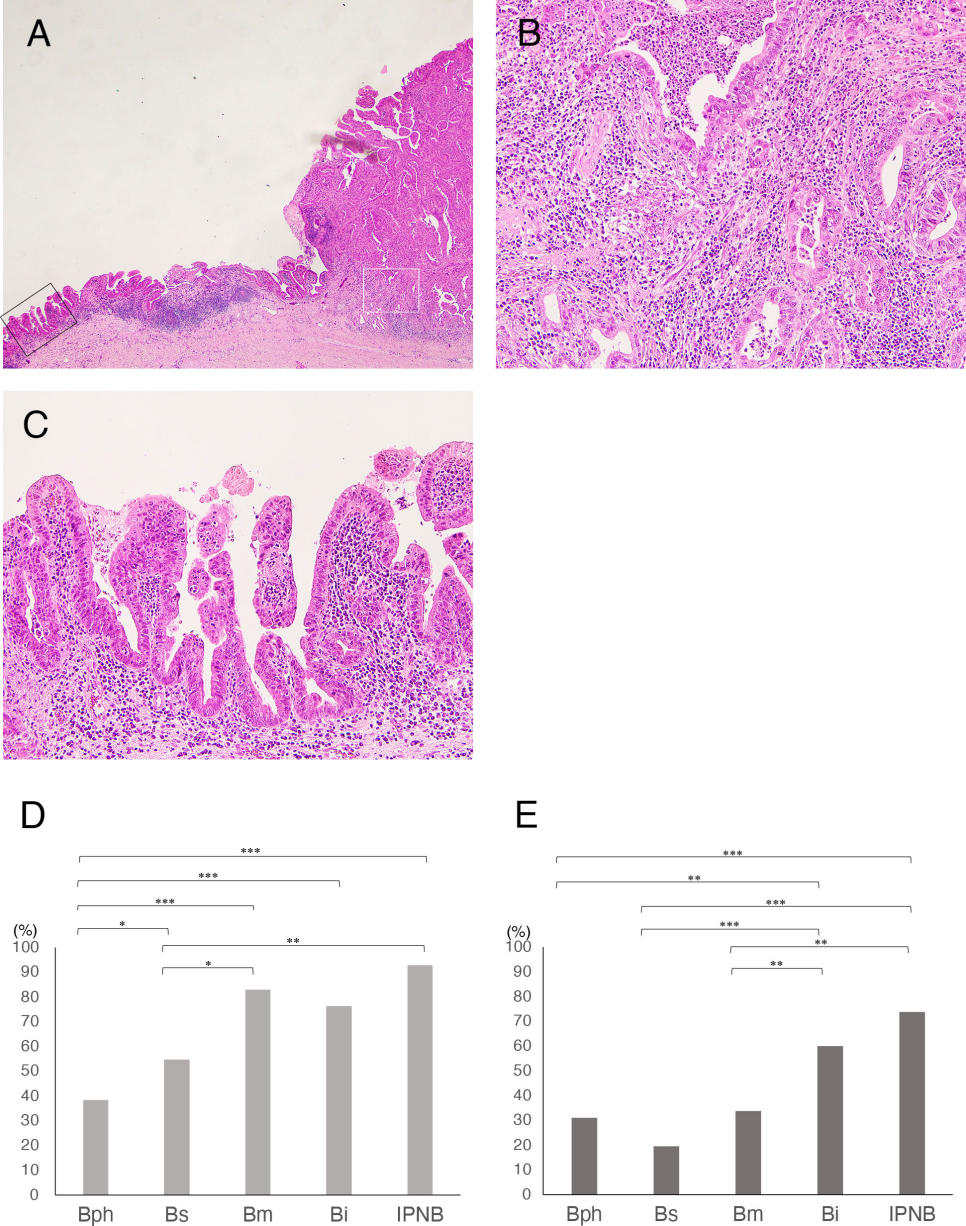
**(A-C)** Histology of intraepithelial spread (IES) of cancer cells in extrahepatic cholangiocarcinomas (eCCAs). **(A)** Main tumor mass with stromal invasion of cancer cells at right and IES extended along epithelial layers of the bile duct in very low-power view. **(B)** Histology of cancer area with stromal invasion in middle power view, corresponding to white square in A. **(C)** Histology of IES showing proliferation of cancer cells in epithelial layer with a low papillary structure in middle power view, corresponding to black square in A. **(D, E)** Comparison of incidence of intraepithelial spread in invasive eCCAs. **(D)** Bar graph shows incidence of IES along extrahepatic bile duct in invasive eCCAs. **(E)** Bar graph shows incidence of IES along with cystic duct (CyD) in invasive eCCAs that cancer cells reached the junction of CyD. Differences are examined by chi-square test. IPNB is not classified by tumor location because of small number of cases. *P< 0.05; **P< 0.01; ***P< 0.001.

IES was observed in 60.2% of invasive eCCAs (**Figure 1**). The incidence of IES in Bph or Bs eCCAs was significantly lower than in Bm or Bi eCCAs or invasive IPNBs (**Figure 1D**). **Table 2A** summarizes the extent and distribution of IES in invasive eCCAs. Invasive IPNBs had the highest incidence (*P <*0.001) and longer duration of IES than invasive conventional eCCAs. The incidence peak of IES was at lengths of ≥10 and <20 mm in conventional eCCAs and at lengths of ≥20 and <30 mm in invasive IPNBs. The IES extended further to the liver-side in invasive eCCAs.

**Table 2A:** Intraepithelial spread of extrahepatic cholangiocarcinomas along with extrahepatic bile duct.

	Bph eCCA n= 96	Bs eCCA n= 42	Bm eCCA n= 59	Bi eCCA n= 59	invasive IPNB n= 28
IES, mm					
0	62 (64.6)	19 (45.2)	13 (22.0)	16 (27.1)	3 (10.7)
0 <, < 10	12 (12.5)	6 (14.3)	10 (16.9)	6 (10.2)	3 (10.7)
10 ≤, < 20	12 (12.5)	9 (21.4)	19 (32.2)	22 (37.3)	7 (25.0)
20 ≤, < 30	5 (5.2)	5 (11.9)	8 (13.6)	7 (11.9)	9 (32.1)
30 ≤, < 40	3 (3.1)	2 (4.8)	5 (8.5)	6 (10.2)	3 (10.7)
40 ≤, < 50	1 (1.0)	0 (0)	3 (5.1)	1 (1.7)	0 (0)
50 ≤, < 60	1 (1.0)	1 (2.4)	0 (0)	0 (0)	3 (10.7)
60 ≤	0 (0)	0 (0)	1 (1.7)	1 (1.7)	0 (0)
Distribution of IES					
liver-side dominant	17 (50.0)	12 (52.2)	18 (39.1)	30 (69.8)	13 (52.0)
duodenal-side dominant	14 (41.2)	10 (43.5)	24 (52.2)	10 (23.3)	8 (32.0)
equivalent	3 (8.8)	1 (4.3)	4 (8.7)	3 (7.0)	4 (16.0)

**Table 2B:** Intraepithelial spread of extrahepatic cholangiocarcinomas along with cystic duct.

	Bph eCCA n= 45	Bs eCCA n= 41	Bm eCCA n= 59	Bi eCCA n= 45	invasive IPNB n= 24
CyD-IES, mm					
0	31 (68.9)	33 (80.5)	39 (66.1)	18 (40.0)	6 (25.0)
0 <, < 10	7 (15.6)	1 (2.4)	5 (8.5)	4 (8.9)	5 (20.8)
10 ≤, < 20	6 (13.3)	3 (7.3)	8 (13.6)	10 (22.2)	6 (25.0)
20 ≤, < 30	1 (2.2)	3 (7.3)	2 (3.4)	7 (15.6)	4 (16.7)
30 ≤	0 (0)	1 (2.4)	5 (8.5)	6 (13.3)	3 (12.5)
Status of IES-CyD					
a part of CyD	11 (78.6)	3 (37.5)	12 (60.0)	19 (70.4)	11 (61.1)
entire CyD	2 (14.3)	4 (50.0)	5 (25.0)	7 (25.9)	3 (16.7)
GB and entire CyD	1 (7.1)	1 (12.5)	3 (15.0)	1 (3.7)	4 (22.2)

Values given are the number of patients (percentage) unless otherwise indicated.

Bi, inferior portion of EHBD; Bm, middle portion of EHBD; Bph, (peri)hilar bile duct; Bs, superior portion of EHBD; CyD, cystic duct; CyD-IES, IES along with CyD; eCCA, extrahepatic cholangiocarcinoma; EHBD, extrahepatic bile duct; GB, gallbladder; IES, intraepithelial spread; IPNB, intraductal papillary neoplasm of the bile duct

**Figure 2:**
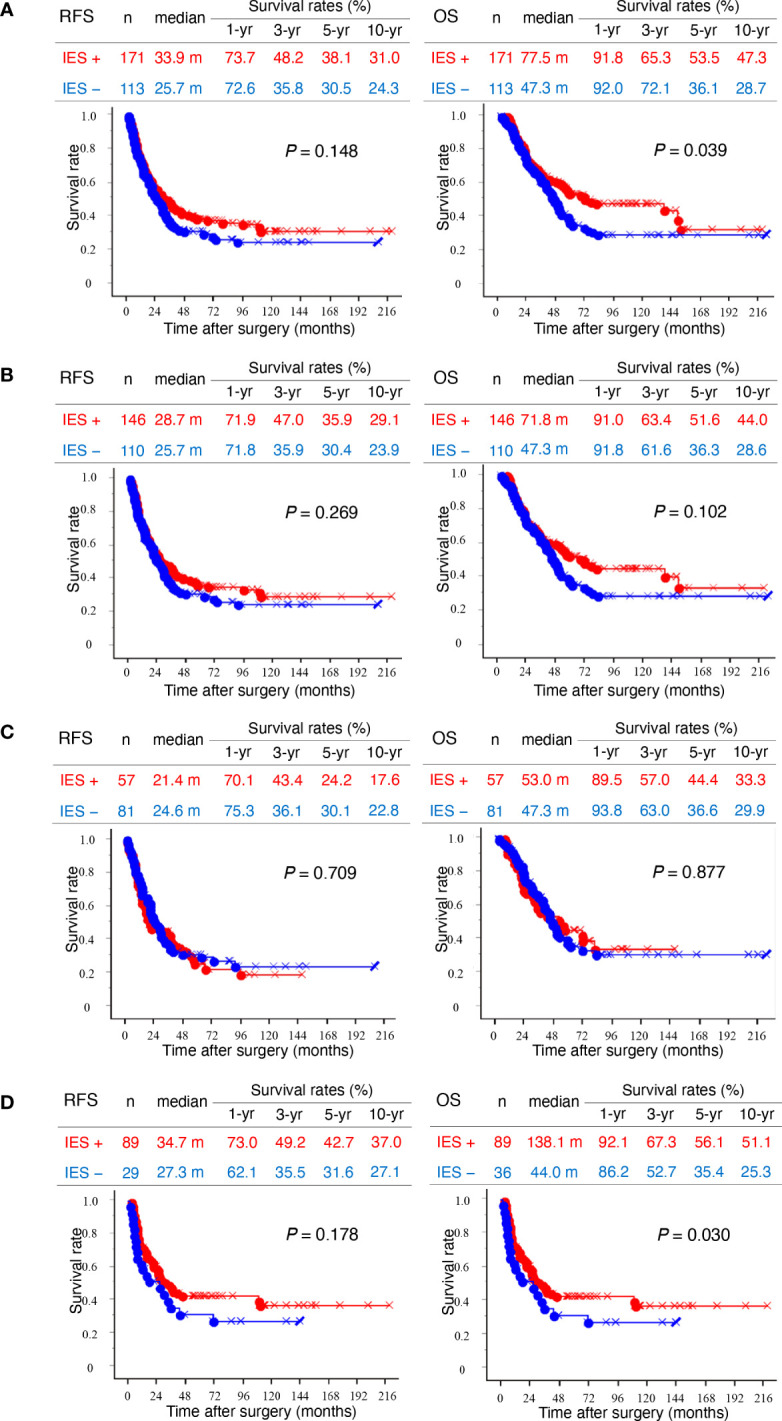
Kaplan–Meier survival curves. **(A)** Left and right panels show recurrence-free survival (RFS) and overall survival (OS), respectively. Kaplan–Meier curves of total invasive extrahepatic cholangiocarcinomas (eCCAs) with intraepithelial spread (IES) (red) and without IES (blue) are compared. **(B)** Kaplan–Meier curves of invasive conventional eCCAs with IES (red) and without IES (blue) are compared. **(C)** Kaplan–Meier curves of perihilar eCCAs with IES (red) and without IES (blue) are compared. **(D)** Kaplan–Meier curves of distal eCCAs with IES (red) and without IES (blue) are compared. Differences are examined by a log-rank test.

Patients with invasive eCCAs and IES showed significantly longer survival times than those without IES (*P* = 0.039) (**Figure 2A**). Similar survival associations were found in patients with invasive conventional eCCAs, but without statistical significance (**Figure 2B**). IES was not associated with outcomes in perihilar eCCAs (**Figure 2C**), although patients with IES showed significantly longer OS in distal eCCAs (*P* = 0.030) (**Figure 2D**). When survival was evaluated at each location in invasive conventional eCCAs and invasive IPNBs, significant differences in RFS (*P* = 0.039) and OS (*P* = 0.025) were observed in Bm eCCAs (**Supplementary Figure 3**). Multivariate analyses of patients with distal eCCAs (**Table 3**) revealed that the IES was not a significant predictor of RFS or OS. In distal eCCAs, the presence of IES significantly correlated with a lower female-to-male ratio, larger total tumor size, higher positive bile duct margins, and a higher positive residual tumor status (**Supplementary Table 2**).

**Table 3:** Univariate and multivariate analysis of conventional extrahepatic cholangiocarcinomas reached to junction of cystic duct (n = 190).

	Recurrence-free survival				Overall survival			
Variables	Univariate		Multivariate		Univariate		Multivariate	
	HR (95% CI)	P value	HR (95% CI)	P value	HR (95% CI)	P value	HR (95% CI)	P value
Age (>69/≤69 years)	0.979 (0.689-1.388)	0.903			0.922 (0.619-1.365)	0.687		
Gender (female/male)	1.293 (0.829-1.948)	0.249			1.498 (0.929-2.329)	0.095		
Tumor location (perihilar/distal)	1.253 (0.881-1.780)	0.208			1.315 (0.889-1.945)	0.170		
Total tumor size (>55/≤55 mm)	1.325 (0.932-1.878)	0.117			1.143 (0.771-1.685)	0.503		
Invasive tumor size (>40/≤40 mm)	1.673 (1.175-2.375)	0.004	1.139 (0.778-1.666)	0.501	1.523 (1.026-2.250)	0.037	1.028 (0.665-1.582)	0.902
CyD-IES (absence/presence)	1.571 (1.085-2.317)	0.016	1.275 (0.869-1.905)	0.217	1.824 (1.198-2.853)	0.005	1.500 (0.970-2.380)	0.068
Histological grade (G2+G3/G1)	2.153 (1.328-3.722)	0.001	1.893 (1.157-3.295)	0.010	1.982 (1.167-3.639)	0.010	1.767 (1.023-3.285)	0.041
Lymphatic invasion (high/low)	2.288 (1.608-3.272)	<0.001	1.799 (1.180-2.752)	0.006	2.824 (1.900-4.237)	<0.001	1.987 (1.235-3.220)	0.005
Venous invasion (high/low)	2.031 (1.430-2.898)	<0.001	1.243 (0.819-1.894)	0.307	2.558 (1.724-3.835)	<0.001	1.477 (0.930-2.367)	0.997
Perineural invasion (high/low)	2.237 (1.342-4.012)	0.001	1.422 (0.816-2.643)	0.222	2.027 (1.180-3.760)	0.009	1.193 (0.663-2.302)	0.570
Residual tumor status (microscopic residual tumor/no residual tumor)	1.941 (1.357-2.804)	<0.001	1.764 (1.194-2.629)	0.004	1.612 (1.090-2.405)	0.017	1.487 (0.960-2.318)	0.076
Lymph node metastasis (presence/absence)	2.272 (1.591-3.272)	<0.001	1.321 (0.878-2.000)	0.182	2.527 (1.698-3.809)	<0.001	1.117 (0.913-2.308)	0.117

CyD, cystic duct; CyD-IES, IES along with CyD; IES, intraepithelial spread.

### Clinicopathological characteristics of invasive eCCAs with IES along CyD

The IES on the EHBD reached the junction of the CyD and often extended beyond the junction and continuously into both the CyD and the other side of the EHBD. When invasive eCCAs in which cancer cells reached the junction of the CyD were selected and assessed, the incidence of IES along with CyD (CyD-IES) was lower than that of IES on EHBD in all locations of the conventional and invasive eCCAs (**Figure 1D** and **Table 2B**). 60.0% of Bi eCCAs had CyD-IES and 30% of the other conventional eCCAs had CyD-IES, and their CyD-IES extended rarely to the gallbladder (**Table 2B**). In contrast, 75.0% of IPNBs had CyD-IES, and 57.1% had IES on the gallbladder when IPNBs reached the border between the CyD and the gallbladder.

**Figure 3:**
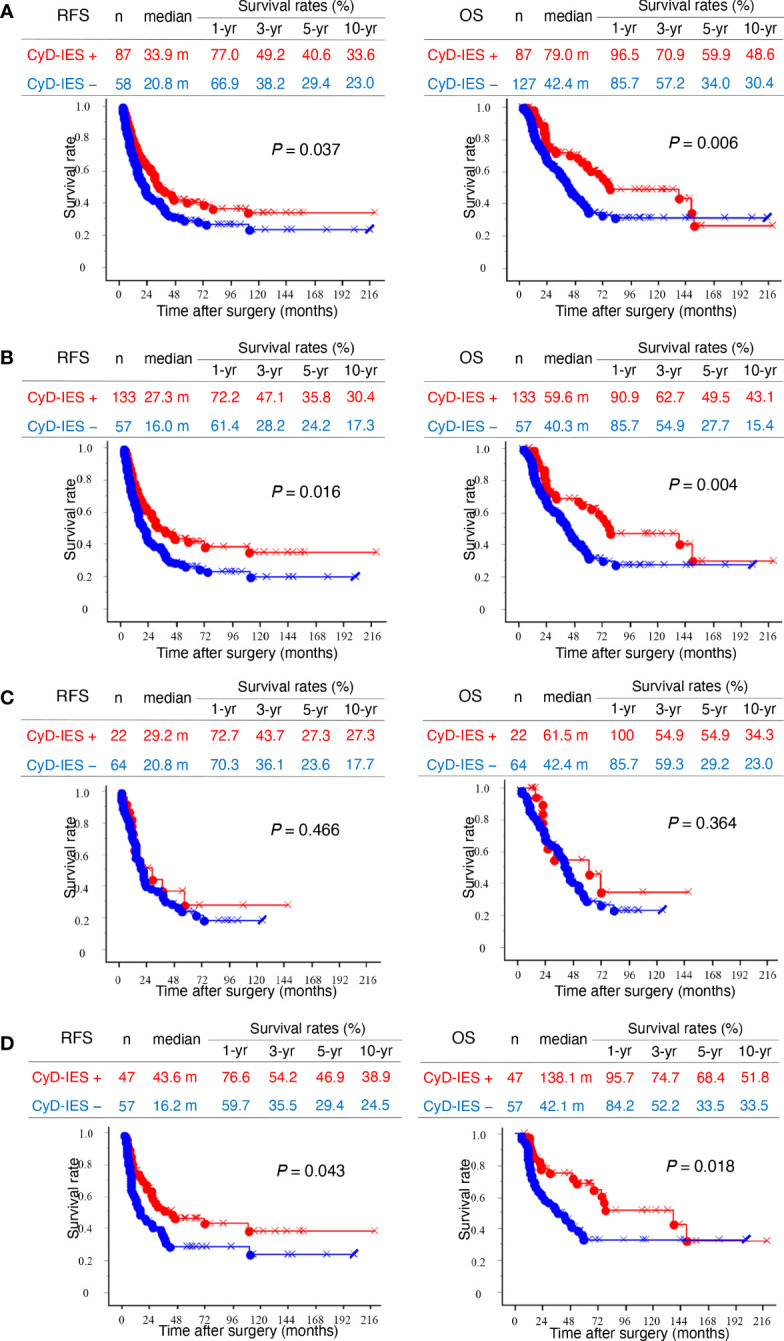
Kaplan–Meier survival curves. **(A)** Left and right panels show recurrence-free survival (RFS) and overall survival (OS), respectively. Kaplan–Meier curves of total invasive extrahepatic cholangiocarcinomas (eCCAs) with intraepithelial spread along with cystic duct (CyD-IES) (red) and without CyD-IES (blue) are compared. **(B)** Kaplan–Meier curves of invasive conventional eCCAs with CyD-IES (red) and without CyD-IES (blue) are compared. **(C)** Kaplan–Meier curves of perihilar eCCAs with CyD-IES (red) and without CyD-IES (blue) are compared. **(D)** Kaplan–Meier curves of distal eCCAs with CyD-IES (red) and without CyD-IES (blue) are compared. Differences are examined by a log-rank test.

In patients with invasive eCCAs, as well as those with invasive conventional eCCAs, patients with CyD-IES showed significantly longer survival times for both RFS (*P* = 0.037 and *P* = 0.016) and OS (*P* = 0.006 and *P* = 0.004, respectively) than patients without CyD-IES (**Figures 3A, B**). Perihilar eCCAs had a low incidence of CyD-IES, and there were no associations between CyD-IES and patient outcomes (**Figure 3C**). Patients with CyD-IES in distal eCCAs had significantly longer survival than those without CyD-IES for both RFS (*P* = 0.043) and OS (*P* = 0.018) (**Figure 3**). Multivariate survival analysis of patients with invasive conventional eCCAs revealed that the CyD-IES was not a significant predictor of RFS or OS (**Table 3**). Multivariate survival analysis of patients with distal eCCAs also revealed that the CyD-IES was not a significant predictor of RFS or OS (**Supplementary Table 3**). CyD-IES closely correlated with tumor location, perineural invasion, and early recurrence of distal eCCAs (**Supplementary Table 4**).

Distal eCCA cases reaching the junction of the CyD were divided into three groups according to IES patterns: eCCAs with CyD-IES as group A, eCCAs with only IES as group B, and eCCAs without any IES or CyD-IES as group C (**Supplementary Figure 4**). Group A contained 5 eCCAs with only CyD-IES and 42 cases with both CyD-IES and IES. Survival analyses revealed that group A had longer survival and group C had shorter survival, with significant differences in both RFS (*P* = 0.002) and OS (*P* < 0.001) (**Supplementary Figure 5**). Group B also showed longer survival compared with that of group C for both RFS (*P* = 0.024) and OS (*P* = 0.034) (**Supplementary Figure 5**). All survival rates were higher in group A than in group B.

### Incidence and profiles of IES in BTCs; IES in eCCAs extended into IHBD and ampullary area

**Figure 4:**
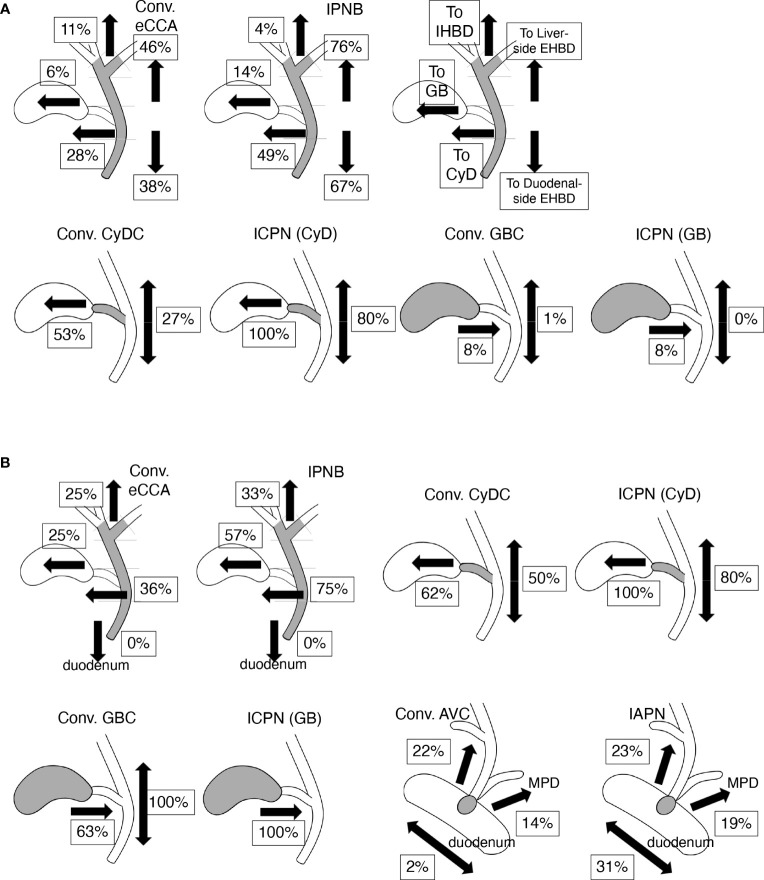
Incidence and profiles of intraepithelial spread (IES) of bile tract cancers (BTCs). Values indicate percentages of IES in each tissue and each direction on biliary tract. Values represent incidence that ratio of positive case among total cases in **(A)** and values represent incidence ratio of positive case to cases in which cancer cells reach tissue border closest to the assessed IES (e.g., the junction of CyD for assessing IES on EHBD in CyDCs) in **(B)**. **(A)** The incidence and profiles of IES in conventional (conv.) extrahepatic cholangiocarcinomas (eCCAs) and intraductal papillary neoplasms of the bile duct (IPNBs) are shown in upper line. Incidence of IES in liver- or duodenal-side on extrahepatic bile duct (EHBD) is shown on right side in each panel. Incidences of IES on intrahepatic bile duct (IHBD), IES on gallbladder (GB), and IES on cystic duct (CyD) are shown on the left side in each panel. Incidence of IES in the gallbladder and EHBD in conventional (conv.) cystic duct cancers (CyDCs) or intracholecystic papillary neoplasms (ICPNs) arising from CyD are depicted in the lower line. Incidence of IES in CyD and EHBD in the conventional (conv.) gallbladder cancers (GBCs) or ICPNs arising in gallbladder are also found on the lower line. **(B)** The incidence of IES extended to various tissues other than the tissue in which the tumors developed in BTCs. The incidence values of IES beyond the ampullary area, including IES on the EHBD, IES on main pancreatic duct (MPD), intraepithelial spread into the duodenum in conventional ampullary cancers (conv. AVCs), and intraampullary papillary tubular neoplasms (IAPNs) are shown in the lower right panels.

Some properties of the IES on the EHBD of the eCCAs are described above and depicted in **Figure 4**. Liver-side IES was ended on the IHBD in 60.4%, on the bile ducts of the borderline area between the IHBD and EHBD (i.e., hepatic ducts and the second branches) in 37.5%, and on the confluence of the right and left hepatic ducts in 2.1% of conventional eCCA cases who underwent hepatectomy with liver-side IES (**Table 4A**). In perihilar eCCAs, IES on EHBD was mostly ended on the EHBD in front of the IHBD, and extension of IES into the IHBD from the EHBD was very rare, whereas 95.5% of cases with IES on IHBD had stromal invasion in the IHBD area. In distal eCCAs, cases of IES on IHBD were rare, with longer IES. Liver-side IES ended on the IHBD in 12.5% of patients, on the confluence of the right and left hepatic ducts in 25.0%, and on EHBD distal to the confluence of the hepatic ducts in 37.5% of patients with IPNB who underwent hepatectomy with liver-side IES. When cancer cells reached the EHBD in front of the IHBD, IES on the IHBD was found in 61.7% of patients. Thus, the liver-side IES from the EHBD often ended in front of the IHBD and extended to the IHBD in a limited conventional eCCAs with a long length of IES or in a part of perihilar eCCAs with stromal invasion of the IHBD.

**Table 4A:** Intraepithelial spread of extrahepatic cholangiocarcinomas directed to intrahepatic bile ducts.

		perihilar eCCA cases undertaken hepatectomy with liver-side IES (n=39)	distal eCCA cases undertaken hepatectomy with liver-side IES (n=9)	invasive IPNB cases undertaken hepatectomy with liver-side IES (n=8)
liver-side end position of IES				
at IHBD		22*	7	1
at EHBD in front of IHBD	around the confluence of and on the second branches **	8	2	0
	hepatic ducts	8	0	2
at confluence of right and left hepatic ducts		1	0	1
at EHBD distal to the confluence of hepatic ducts	Bs	0	0	4

*containing 21 cases with stromal invasion reached to IHBD area and one case with IES extended from Bs. **right posterior or anterior sectoral ducts or segmental ducts of 2, 3, or 4. Bs, superior portion of EHBD; eCCA, extrahepatic cholangiocarcinoma; EHBD, extrahepatic bile duct; IES, intraepithelial spread; IHBD, intrahepatic bile duct; IPNB, intraductal papillary neoplasm of the bile duct.

**Table 4B:** Intraepithelial spread of extrahepatic cholangiocarcinomas directed to ampullary area.

		perihilar eCCA cases undertaken pancreatoduodenectomy with duodenal-side IES (n=8)	distal eCCA cases undertaken pancreatoduodenectomy with duodenal-side IES (n=56)	IPNB cases undertaken pancreatoduodenectomy with duodenal-side IES (n=13)
duodenal-side end position of IES				
at duodenum		0	0	0
at ampullary area	ampullary common duct	0	13	4
	ampullary bile duct	2	25	6
at EHBD	Bi	6	18	3

Bi, inferior portion of EHBD; eCCA, extrahepatic cholangiocarcinoma; EHBD, extrahepatic bile duct; IES, intraepithelial spread; IHBD, intrahepatic bile duct; IPNB, intraductal papillary neoplasm of the bile duct.

Duodenal-side IES ended in the ampullary common duct in 20.3%, in the ampullary bile duct in 42.2%, and in 37.5% of patients with conventional eCCA who underwent pancreatoduodenectomy with duodenal-side IES (**Table 4B**). Duodenal-side IES ended in the ampullary common duct in 30.8%, in the ampullary bile duct in 46.2%, and in 23.1% of patients with IPNB who underwent pancreatoduodenectomy with duodenal-side IES. Thus, the duodenal-side IES on EHBD and the ampullary duct were similar to one tissue without barriers, although it did not reach the duodenum.

### IES in CyDCs

The incidence of IES in CyDCs was 80.0%; IES on CyD was found in 25.0% of cases; IES on the gallbladder in 65.0%; IES on EHBD in 40.0% (liver-side in 30.0% and duodenal-side in 15.0%); and no IES on the IHBD, ampullary common duct, or duodenum. The IES from CyDCs extended in both directions of the gallbladder and EHBD in 40.0% of the cases. The incidence of IES in conventional CyDCs and ICPNs is shown in **Figure 4** and **Supplementary Table 5**. CyDCs had a high frequency of IES on both the gallbladder and EHBD, especially in ICPN showing a very high frequency of IES; their incidences were 53% and 27% in conventional CyDCs and 100% and 80% in ICPNs, respectively.

### IES of GBCs

IES on the gallbladder was often observed in GBCs, although IES extending into the CyD and EHBD was not observed, which was found in 8.1% and 1.2% of GBCs, respectively. This series contained 30.2% ICPN cases, and ICPNs showed a similar incidence of IES on CyD as conventional GBCs, as shown in **Figure 4A** and **Supplementary Table 5**. However, in conventional GBCs and ICPNs that reached the borders between the CyD and the gallbladder, IES on the CyD was found in 62.5% and 100% of cases, respectively (**Figure 4B**). Thus, GBCs often have an IES, although the IES extension is usually limited to the gallbladder. The low incidence of IES on the CyD in both conventional GBCs and ICPNs was presumed to be because the tumor cells did not reach the border between the gallbladder and the CyD.

### IES of AVCs

**Figure 5:**
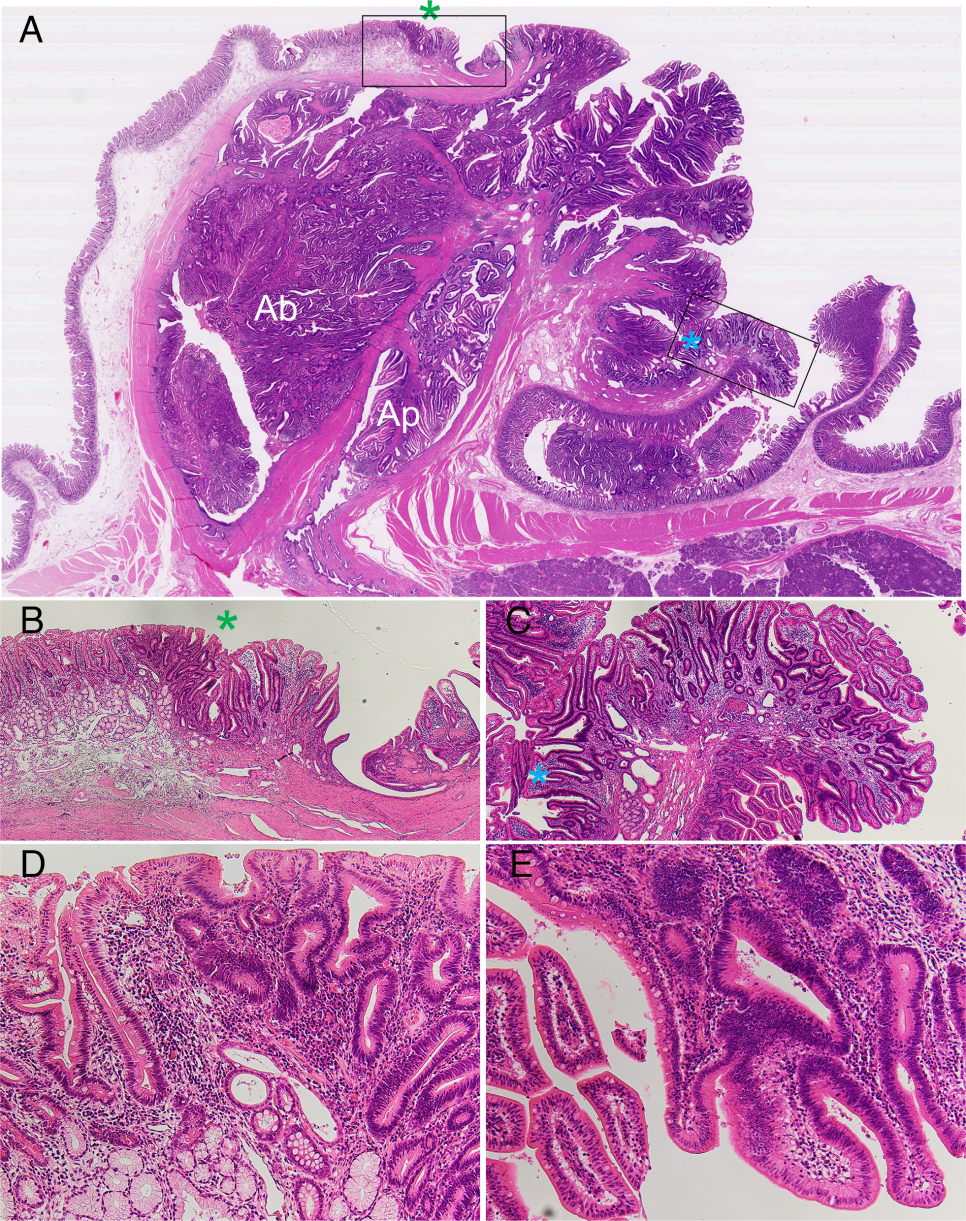
Histology of intraepithelial spread of cancer cells from ampullary common duct to duodenum in ampullary cancer. At an opening of ampullary common duct surrounded by Oddi sphincter, Oddi sphincter anastomoses to muscularis mucosa in duodenal mucosal layer (*). An opening of common duct is covered by epithelial cells of common duct that continue to the duodenal covering epithelium on the border of *. The Brunner’s glands are found in the duodenum. Right and left side borders are indicated by blue-colored * and green-colored *. Cancer cells with papillary growth extend along common duct and replace the existing covering epithelium beyond the borders (*). **(A)** is loupe figure; **(B, C)** are in low power view corresponded to left and right squares in **(A)** respectively; **(D, E)** are in middle power view.

The incidence of IES was investigated in AVCs raised in the ampulla of Vater, except for the ampullary duodenum, which was 60.8%, 54.0%, and 76.9% in total AVCs, conventional AVCs, and IAPNs, respectively. Three directions of the IES beyond the ampulla of Vater were found: the EHBD, main pancreatic duct (MPD), and duodenum. The incidences of these IES are shown in **Figure 4B** and **Supplementary Table 5**. These IESs beyond the ampulla of Vater were usually of short length (**Supplementary Table 6**); IES on EHBD with ≤5 mm in length was in 54.5% of conventional AVCs and in 58.3% of IAPNs; IES on MPD with ≤5 mm in length was in 57.1% of conventional AVCs and in 70.0% of IAPNs. In 98.0% of conventional AVCs, the extension of the IES ended at the border between the ampullary common duct and the duodenum (**Figure 5**), whereas 30.8% of IAPN cases had the IES extending beyond the border into the duodenum (**Figure 4B**). There was no IES reaching the IHBD, CyD, or gallbladder, except in one case of IAPN that spread to the CyD. Thus, IES extensions into the EHBD and MPD were found in approximately 20% and 15% of cases, respectively, and short length in both conventional AVCs and IAPNs, although intraepithelial spread on the duodenum was found in approximately 1/3 of IAPNs but not in conventional AVCs.

## Discussion

eCCAs are rare and show aggressive behaviors (2), and their clinicopathological characteristics vary depending on their anatomical location (7–10). IES may be a hallmark of one type of extension in cancer biology, in which intraepithelial extension of cancer cells may be predominant over stromal invasion. Hence, stromal invasion is relatively weak in case of IES. This study revealed that the presence of IES was associated with favorable outcomes in eCCAs, although this was dependent on tumor location and type. The incidence and extension properties of IES are also characterized by the tumor location and type. Compared to conventional eCCAs, invasive IPNBs showed a high incidence and a longer extension of IES, often spreading beyond the tissue borders. Invasive IPNBs with IES were also associated with good prognosis. In conventional eCCAs, the incidence of IES was significantly higher in distal eCCAs than in perihilar eCCAs, although the length of the IES extension was comparable (**Figure 1**). The presence of IES was associated with better outcomes in conventional eCCAs and a significantly longer survival time for OS in distal eCCAs (**Figure 2**). The presence of CyD-IESs was significantly associated with better outcomes in total invasive eCCAs, invasive conventional eCCAs, and distal eCCAs. The incidence of CyD-IES was higher in distal eCCAs than in perihilar eCCAs, which was reduced to 35–80% of that of IES in each conventional eCCAs. CyD-IES and IES often overlap in the same eCCA cases, and the CyD-IES could more effectively stratify eCCAs to predict patient outcomes. When invasive eCCAs reach the junction of the CyD, CyD-IES is a more useful prognostic factor than IES. It is implied that clinicopathological characteristics are apparently different between perihilar eCCAs and distal eCCAs, and further among Bph, Bs, Bm, and Bi eCCAs, the differences may be due to not only simple location differences but also the biological properties of cancer cells.

The incidence and extension properties of IES also differ depending on the different types of BTCs. All BTCs showed common characteristics in that the incidence of IES was more than a half in tissues that the tumor raised, although IES extension to other tissues beyond the borders decreased the incidence. In addition to these common rules, the incidence and properties of IES differed depending on the tumor location and type (**Figure 4**). These properties may be useful for determining the primary sites of BTCs.

The presence of IES was a favorable prognostic factor in patients with conventional eCCA in this series. The extensive IES defined as more than 20 mm in length beyond the invasive area, is associated with better patient outcome in eCCAs in the previous reports (11, 13, 15). The extensive IES (≥ 20 mm) was not prognostic in our series, even if the cohort was combined with conventional eCCAs and invasive IPNBs, or was divided by anatomical locations. This discrepancy may be due to the cohort used in the present study. In previous studies (11, 13), researchers have analyzed invasive eCCAs combined with conventional eCCAs and invasive IPNBs, and such cohorts had much higher rates of patients with perihilar eCCAs (74.5% and 82.6%, respectively) than those in this study (51.4%). Since the incidence of IES-positive cases in perihilar eCCAs was low, most of the IES-positive cases in previous studies were invasive IPNBs, and IES-negative cases were conventional perihilar eCCAs; therefore, the differences in patient outcomes might be significant.

In this study, Bph eCCAs showed unique clinicopathological characteristics compared to other eCCAs. Patients with Bph eCCAs tended to be younger females. Compared to eCCAs, intrahepatic cholangiocarcinoma (iCCA) develops predominantly in males but is more common in females, and the peak incidence age in iCCA is approximately ten years younger than that in eCCAs (28). It is suggested that Bph eCCAs might be similar to iCCAs. Bph eCCAs had much more venous invasion, together with a low incidence of IES in perihilar eCCAs, which is consistent with the aggressive behavior and poor outcomes of perihilar eCCAs. In addition, Bph eCCAs showed unique features in the incidence and properties of IES, especially IES on IHBD, compared to other conventional eCCAs. In contrast, Bi eCCAs showed lower perineural invasion and a higher incidence of IES and CyD-IES, with a better prognosis, similar to that of invasive IPNBs (**Supplementary Figure 1**).

The biliary tree differentiates from a few different anlagen raised in the hepatic diverticulum during embryonic development; the proximal part of the hepatic ducts and intrahepatic bile ducts are developed from the ductal plate that appears in the hepatic hilus in the developing liver; the distal part of hepatic ducts, common hepatic duct, and common bile duct are formed from the caudal part of the liver bud, and the gallbladder with the cystic duct is formed from the gallbladder anlage (29–31). These tissue borders based on embryonic development almost correspond to the extension of IES in BTCs, with some tissue-dependent modifications.

The border between the IHBD and EHBD is sometimes difficult to determine because there are many variations in bile duct branching, including in this area. Certain IHBD are the liver side of the third branch of the bile duct (e.g., segmental ducts 5 and 8), and certain EHBD are the duodenal side of the common hepatic duct, while it is possible that the border between the IHBD and EHBD might vary by individual within the perihilar bile ducts between them. Most endpoints of the liver-side IES were found in this area (**Table 4A**). It was assumed that the endpoints of the liver-side IES represented the border. The incidence of IES in perihilar eCCAs was lower, and IES in IHBD was rare when IES extended from the EHBD to the IHBD in conventional eCCAs. In contrast, most perihilar eCCA with IES on IHBD had stromal invasion of the IHBD area without IES on EHBD. It is possible that some of these perihilar eCCAs developed from the IHBD and extended from the IES to the IHBD.

The duodenal-side IES extended on the EHBD and ampullary ducts, as they are one tissue without potential extension barriers. This might be reasonable because both ducts develop into one tube in the embryonic developmental stage. The actual duodenal-side tissue border should be at the border between the ampullary common duct and the duodenum. The IES of conventional AVCs that develop in the common duct or ampullary bile duct usually ends at the common duct-duodenal border, and in 30% of IAPNs, the intraepithelial spread extends beyond the border into the duodenum.

CyD develops from the gallbladder anlage during the embryonic stage, and the histological structure of CyD is the same as that of EHBD, but different from that of the gallbladder, suggesting that CyD is similar to a zone of brackish water. CyD may overlap biologically (i.e., molecular expression) with EHBD and the gallbladder. The incidence of IES in the gallbladder from conventional CyDCs that reached the border between the gallbladder and CyD and the incidence of IES in the CyD from conventional GBCs that reached the border were almost the same (62% and 63%, respectively) (**Figure 4B**). Similarly, the incidence of IES in the gallbladder from the ICPNs of the CyD and that of IES in CyD from the ICPNs of the gallbladder were both 100%. It is suggested that the CyD and the gallbladder might be recognized as similar tissues by GBCs and CyDCs with respect to IES extension, as represented by the developmental event in which these tissues develop from the gallbladder anlage. Fifty percent of conventional CyDCs that reached the junction of the CyD had an IES on the EHBD, and 36% of conventional eCCAs reached the junction of the CyD with the CyD-IES. This difference in incidence might be attributed to the difference in distances from the invasive cancers to the junction of the CyD.

IPNB, ICPN, and IAPN are thought to be counterpart entities of pancreatic intraductal papillary mucinous neoplasms. The extension property of IES from ICPNs is similar to that of IPNBs, in which IES often extends beyond tissue borders compared to conventional types of cancers and has a relatively longer length. However, ICPNs spread more frequently beyond the border between the gallbladder and CyD compared to IPNBs. This difference may be explained by the distance from the main tumor. Similarly, both IPNBs and ICPNs of the CyD extended the IES frequently *via* the junction of the CyD, although the ICPNs of the gallbladder did not reach the junction. In contrast, IAPNs revealed different characteristics from IPNBs and ICPNs in the incidence and extension properties of IES, which did not have a longer IES. IAPNs had similar properties to conventional AVCs in IES for EHBD and MPD. However, IAPNs showed an apparently higher frequency of intraepithelial spread into the duodenum compared to conventional AVCs. It is necessary to characterize these similar entities to establish their positions.

From a clinical standpoint, perioperative chemotherapy is a standard strategy, even for resectable eCCAs and PDACs. The presence of IES revealed by pathological investigation may be an indicator of the postoperative treatment strategy. More importantly, the different incidences of IES and related outcomes suggest biological differences between perihilar and distal eCCAs. Along with integrative molecular profiling analyses, targeted therapies have been developed for advanced eCCAs and iCCAs (32–34). Genetic or molecular alterations in CCAs related to tumor localization and the presence of IES are still limited. Investigating molecular or genetic differences is imperative for identifying clinical features and establishing targeted therapeutic options.

This study has several limitations. First, this was a retrospective study conducted in a single-center cohort. Second, differences in the adapted surgical procedures, including lymph node dissection, could affect survival outcomes. However, this study included a relatively large number of eCCAs, and the therapeutic strategy did not change significantly during the study period. Additionally, a pathological diagnosis was made based on a detailed pathological examination in each case. This consistency in the treatment and diagnosis is an advantage of this study. As there are still few studies investigating IES in eCCAs, further investigation is required to identify IES and their characteristics.

In conclusion, the IES was a favorable factor in patients with eCCA, although it was not as strongly favorable as previously reported. CyD-IES is also a favorable factor and may be more useful for IES when eCCAs reach the CyD junction. The clinicopathological characteristics of eCCAs vary depending on their anatomical location and type, especially between perihilar and distal eCCAs. The incidence and extension properties of IES also differ depending on the different types of BTCs. We hypothesize that the extension profiles of the IES may represent the tumor cell origin as well as the biological characteristics of cancer.

## Data availability statement

The datasets used and analyzed during the current study are available from the corresponding author upon reasonable request. 

## Ethics statement

This study was approved by the Institutional Review Board of National Cancer Center Hospital, Japan (#2016-006). Informed consent was obtained from all patients, and all clinical assessments were conducted in accordance with the principles of the Declaration of Helsinki. 

## Author contributions

Study concept and design: DN and NH. Acquisition of data, analysis, and interpretation of data: DN and NH. Drafting of the manuscript: DN. Critical revision of the manuscript for important intellectual content: ME, SN, DB, TT, TM, KS, and NH. Obtained funding: NH. Technical or material support: ME, SN, DB, TT, TM, KS, and NH. Study supervision, NH. All authors contributed to the article and approved the submitted version.
