# Supplementary material for: Novel insights into the intraepithelial spread of extrahepatic cholangiocarcinoma: clinicopathological study of 382 cases on extrahepatic cholangiocarcinoma
Source: Front Oncol. 2023 Aug 17;13:1216097. doi: 10.3389/fonc.2023.1216097 (PMC10470634; doi:10.3389/fonc.2023.1216097)
Supplement: Supplementary file 1 [file DataSheet_1.docx]

Supplementary Material

**Novel insights into the intraepithelial spread of extrahepatic cholangiocarcinoma: Clinicopathological study of 382 cases on extrahepatic cholangiocarcinoma**

Daisuke Nagashima^1,2,3,4^, Minoru Esaki^2^, Satoshi Nara^2^, Daisuke Ban^2^, Takeshi Takamoto^2^, Takahiro Mizui^2^, Kazuaki, Shimada^2^, Nobuyoshi Hiraoka^1,3,4^

*** Correspondence:** Nobuyoshi Hiraoka, MD, PhD
nhiraoka@ncc.go.jp

Supplementary Table 1. Demography of cases with gallbladder cancers, cystic duct cancers, or ampullary cancers

|  | CyDC, n=20 |  | GBC, n=172 |  | AVC, n=102 |
| --- | --- | --- | --- | --- | --- |
| Age, years [median] | 73.5 [58-84] | Age, years [median] | 69 [41-87] | Age, years [median] | 68.5 [35-90] |
| Female/ Male | 7/ 13 | Female/ Male | 78/ 94 | Female/ Male | 41/ 61 |
| ICPN | 4 (25.0) | ICPN | 52 (30.2) | IAPN | 52 (51.0) |
|  |  | Main location (F/B/N) | 103/ 41 / 28 |  |  |
| TNM classification |  | TNM classification |  | TNM classification |  |
| T category |  | T category |  | T category |  |
| is | 2 (10.0) | is | 23 (13.4) | is | 20 (19.6) |
| 1a | 0 (0) | 1a | 1 (0.6) | 1a | 12 (11.8) |
| 1b | 0 (0) | 1b | 2 (1.2) | 1b | 17 (16.7) |
| 2 | 11 (55.0) | 2a | 30 (17.4) | 2 | 23 (22.5) |
|  |  | 2b | 24 (14.0) |  |  |
| 3 | 3 (15.0) | 3 | 67 (39.0) | 3a | 18 (17.6) |
|  |  |  |  | 3b | 12 (11.8) |
| 4 | 4 (20.0) | 4 | 25 (14.5) | 4 | 0 (0) |
| N category |  | N category |  | N category |  |
| 0 | 13 (65.0) | 0 | 93 (54.1) | 0 | 66 (64.7) |
| 1 | 5 (25.0) | 1 | 51 (29.7) | 1 | 26 (25.5) |
| 2 | 2 (10.0) | 2 | 28 (16.3) | 2 | 10 (9.8) |
| M category |  | M category† |  | M category |  |
| 0 | 20 (100) | 0 | 167 (97.1) | 0 | 100 (100) |
| 1 | 0 (0) | 1 | 5 (2.9) | 1 | 0 (0) |

†All the metastases were liver metastases identified in the resected specimen.

AVC, ampullary cancer; CyDC, cystic duct cancer; F/B/N: fundus/body/neck; GBC, gallbladder cancer; IAPN, intraampullary papillary tubular neoplasm; ICPN, intracholecystic papillary neoplasm.

Supplementary Table 2. Correlation analysis of distal extrahepatic cholangiocarcinomas (n = 118).

|  | IES+ (n= 89) | IES− (n= 29) | P value |
| --- | --- | --- | --- |
| Age, year | 70.0 [41-83] | 69.0 [55-83] | 0.783 |
| Female/ Male | 11/ 78 | 11/ 18 | 0.002 |
| Main tumor location |  |  | 0.521 |
| Bm | 46 (51.7) | 13 (44.8) |  |
| Bi | 43 (48.3) | 16 (55.2) |  |
| Total tumor size, mm | 60 [20-120] | 35 [15-70] | < 0.001 |
| Invasive tumor size, mm | 35 [15-70] | 35 [15-70] | 0.920 |
| Histology |  |  | 0.476 |
| Tub1 | 17 (19.1) | 3 (10.3) |  |
| Tub2 | 48 (53.9) | 19 (65.5) |  |
| Por | 19 (21.4) | 6 (20.7) |  |
| Pap | 4 (4.5) | 0 (0) |  |
| AS | 1 (1.1) | 1 (3.5) |  |
| Depth of invasion |  |  | 0.246 |
| fm | 0 (0) | 1 (3.5) |  |
| ss | 89 (100) | 28 (96.5) |  |
| si | 0 (0) | 0 (0) |  |
| se | 0 (0) | 0 (0) |  |
| Lymphatic invasion |  |  | 0.198 |
| high | 43 (48.3) | 18 (62.1) |  |
| low | 46 (51.7) | 11 (37.9) |  |
| Venous invasion |  |  | 0.166 |
| high | 36 (40.5) | 16 (55.2) |  |
| low | 53 (59.5) | 13 (44.8) |  |
| Perineural invasion |  |  | 0.823 |
| high | 72 (80.9) | 24 (82.8) |  |
| low | 17 (19.1) | 5 (17.2) |  |
| Bile duct margin |  |  | 0.007 |
| presence with invasive cancer | 8 (9.0) | 2 (6.9) |  |
| presence with non-invasive cancer | 28 (31.5) | 1 (3.5) |  |
| absence | 53 (59.6) | 26 (89.7) |  |
| Residual tumor status |  |  | 0.012 |
| microscopic residual tumor | 42 (47.2) | 6 (20.7) |  |
| no residual tumor | 47 (52.8) | 23 (79.3) |  |
| Recurrence |  |  | 0.265 |
| presence | 51 (57.3) | 20 (69.0) |  |
| absence | 38 (42.7) | 9 (31.0) |  |
| Early recurrence |  |  | 0.249 |
| presence | 25 (28.1) | 12 (41.4) |  |
| absence | 64 (71.9) | 17 (58.6) |  |
| TNM classification |  |  |  |
| T category |  |  | 0.084 |
| T1 | 23 (25.8) | 3 (10.3) |  |
| T2 | 54 (60.7) | 18 (62.1) |  |
| T3 | 12 (13.5) | 8 (27.6) |  |
| T4 | 0 (0) | 0 (0) |  |
| N category |  |  | 0.303 |
| N0 | 48 (53.9) | 13 (44.8) |  |
| N1 | 30 (33.7) | 9 (31.0) |  |
| N2 | 11 (12.4) | 7 (24.1) |  |
| M category |  |  | 0.644 |
| M0 | 84 (94.4) | 28 (96.5) |  |
| M1 | 5 (5.6) | 1 (3.5) |  |
| Stage |  |  | 0.421 |
| I | 14 (15.7) | 2 (6.9) |  |
| IIA | 36 (40.5) | 10 (34.5) |  |
| IIB | 25 (28.1) | 10 (34.5) |  |
| IIIA | 9 (10.1) | 6 (20.7) |  |
| IIIB | 0 (0) | 0 (0) |  |
| IV | 5 (5.6) | 1 (3.5) |  |

Values given are the number of patients (percentage) unless otherwise indicated.

AS, adenosquamous carcinoma; Bi, inferior portion of EHBD; Bm, middle portion of EHBD; CyD-IES, IES on cystic duct; EHBD, extrahepatic bile duct; fm, fibromusclar layer; IES, intraepithelial spread; Pap, papillary adenocarcinoma; Por, poorly differentiated adenocarcinoma; se, exposed on serosal surface; si, infiltration beyond the serosa to other tissues; ss, subserosal tissue; Tub1, well differentiated tubular adenocarcinoma; Tub2, moderately differentiated tubular adenocarcinoma.

Supplementary Table 3. Univariate and multivariate analysis of distal extrahepatic cholangiocarcinomas reached to junction of cystic duct (n = 104).

|  | Recurrence-free survival | | | | Overall survival | | | |
| --- | --- | --- | --- | --- | --- | --- | --- | --- |
| Variables | Univariate |  | Multivariate |  | Univariate |  | Multivariate |  |
|  | HR (95% CI) | P value | HR (95% CI) | P value | HR (95% CI) | P value | HR (95% CI) | P value |
| Age (>70/ ≤70 years) | 1.209 (0.742-1.968) | 0.444 |  |  | 1.057 (0.600-1.833) | 0.845 |  |  |
| Gender (female/ male) | 1.562 (0.832-2.743) | 0.157 |  |  | 1.932 (0.991-3.524) | 0.053 |  |  |
| Tumor location (Bm/ Bi) | 1.487 (0.910-2.481) | 0.114 |  |  | 1.808 (1.030-3.302) | 0.039 | 1.720 (0.902-3.382) | 0.100 |
| Total tumor size (>50/ ≤50 mm) | 1.095 (0.669-1.835) | 0.722 |  |  | 0.963 (0.946-2.872) | 0.894 |  |  |
| Invasive tumor size (>35/ ≤35 mm) | 1.956 (1.198-3.232) | 0.007 | 1.462 (0.848-2.547) | 0.172 | 1.642 (0.946-2.872) | 0.078 |  |  |
| IES (absence/ presence) | 2.004 (1.120-3.415) | 0.021 |  |  | 2.464 (1.329-4.367) | 0.005 |  |  |
| CyD-IES (absence/ presence) | 1.660  (1.017-2.754) | 0.042 | 1.162 (0.693-1.977) | 0.571 | 1.953 (1.122-3.501) | 0.018 | 1.190 (0.639-2.251) | 0.585 |
| Histological grade (G2+G3/ G1) | 1.793 (0.957-3.740) | 0.070 |  |  | 1.703 (0.848-3.907) | 0.141 |  |  |
| Depth of invasion (>7/ ≤7 mm) | 2.109 (1.283-3.546) | 0.003 | 1.791 (1.056-3.106) | 0.030 | 1.956 (1.128-3.466) | 0.017 | 1.726 (0.931-3.254) | 0.083 |
| Lymphatic invasion (high/ low) | 2.613 (1.589-4.396) | < 0.001 | 1.749 (0.989-3.130) | 0.055 | 2.886 (1.639-5.258) | < 0.001 | 2.128 (1.108-4.197) | 0.023 |
| Venous invasion (high/ low) | 1.513 (0.928-2.456) | 0.096 |  |  | 1.955 (1.136-3.383) | 0.016 | 1.060 (0.563-2.015) | 0.857 |
| Perineural invasion (high/ low) | 2.193 (1.136-4.771) | 0.018 | 1.186 (0.577-2.701) | 0.657 | 1.873 (0.938-4.181) | 0.077 |  |  |
| Residual tumor status (microscopic residual tumor/ no residual tumor) | 1.969 (1.208-3.241) | 0.007 | 1.468 (0.854-2.542) | 0.165 | 1.723 (1.000-2.986) | 0.050 | 1.366 (0.756-2.485) | 0.301 |
| Lymph node metastasis (presence/ absence) | 2.723 (1.662-4.535) | < 0.001 | 1.941 (1.128-3.406) | 0.016 | 2.909 (1.669-5.224) | < 0.001 | 1.880 (1.011-3.593) | 0.046 |

CyD, cystic duct; CyD-IES, IES along with CyD; IES, intraepithelial spread

Supplementary Table 4. Correlations between intraepithelial spread along with cystic duct and various clinicopathological factors in distal extrahepatic cholangiocarcinomas (n = 104).

|  | CyD-IES+ (n= 47) | CyD-IES− (n= 57) | P value |
| --- | --- | --- | --- |
| Age, year | 68.0 [41-83] | 71 [44-82] | 0.454 |
| Female/ Male | 7/ 40 | 12/ 45 | 0.419 |
| Main tumor location |  |  | 0.008 |
| Bm | 20 (42.6) | 39 (68.4) |  |
| Bi | 27 (57.4) | 18 (31.6) |  |
| Total tumor size, mm | 60 [15-120] | 55 [20-115] | 0.058 |
| Invasive tumor size, mm | 35 [15-65] | 35 [15-70] | 0.857 |
| Histology |  |  | 0.894 |
| Tub1 | 7 (14.9) | 11 (19.3) |  |
| Tub2 | 26 (55.3) | 33 (57.9) |  |
| Por | 11 (23.4) | 11 (19.3) |  |
| Pap | 2 (4.3) | 1 (1.8) |  |
| AS | 1 (2.1) | 1 (1.8) |  |
| Depth of invasion |  |  | 0.452 |
| fm | 1 (2.1) | 0 (0) |  |
| ss | 46 (97.9) | 57 (100) |  |
| si | 0 (0) | 0 (0) |  |
| se | 0 (0) | 0 (0) |  |
| Lymphatic invasion |  |  | 0.343 |
| high | 22 (46.8) | 32 (56.1) |  |
| low | 25 (53.2) | 25 (43.9) |  |
| Venous invasion |  |  | 0.133 |
| high | 17 (36.2) | 29 (50.9) |  |
| low | 30 (63.8) | 28 (49.1) |  |
| Perineural invasion |  |  | 0.024 |
| high | 34 (72.3) | 51 (89.5) |  |
| low | 13 (27.7) | 6 (10.5) |  |
| Bile duct margin |  |  | 0.187 |
| presence with invasive cancer | 2 (4.3) | 8 (14.0) |  |
| presence with non-invasive cancer | 15 (31.9) | 13 (22.8) |  |
| absence | 30 (63.8) | 36 (63.2) |  |
| Residual tumor status |  |  | 0.478 |
| microscopic residual tumor | 19 (40.4) | 27 (47.4) |  |
| no residual tumor | 28 (59.6) | 30 (52.6) |  |
| Recurrence |  |  | 0.117 |
| presence | 26 (55.3) | 40 (70.2) |  |
| absence | 21 (44.7) | 17 (29.8) |  |
| Early recurrence |  |  | 0.008 |
| presence | 7 (18.4) | 29 (43.9) |  |
| absence | 31 (81.6) | 37 (56.1) |  |
| TNM classification |  |  |  |
| T category |  |  | 0.135 |
| T1 | 12 (25.5) | 14 (24.6) |  |
| T2 | 31 (66.0) | 30 (52.6) |  |
| T3 | 4 (8.5) | 13 (22.8) |  |
| T4 | 0 (0) | 0 (0) |  |
| N category |  |  | 0.275 |
| N0 | 28 (59.6) | 25 (43.9) |  |
| N1 | 13 (27.7) | 21 (36.8) |  |
| N2 | 6 (12.8) | 11 (19.3) |  |
| M category |  |  | 0.406 |
| M0 | 43 (91.5) | 55 (96.5) |  |
| M1 | 4 (8.5) | 2 (3.5) |  |
| Stage |  |  | 0.107 |
| I | 9 (19.2) | 7 (12.3) |  |
| IIA | 21 (44.7) | 18 (31.6) |  |
| IIB | 10 (21.3) | 19 (33.3) |  |
| IIIA | 3 (6.4) | 11 (19.3) |  |
| IIIB | 0 (0) | 0 (0) |  |
| IV | 4 (8.5) | 2 (3.5) |  |

Values given are the number of patients (percentage) unless otherwise indicated.

AS, adenosquamous carcinoma; Bi, inferior portion of EHBD; Bm, middle portion of EHBD; CyD-IES, IES on cystic duct; EHBD, extrahepatic bile duct; fm, fibromusclar layer; IES, intraepithelial spread; Pap, papillary adenocarcinoma; Por, poorly differentiated adenocarcinoma; se, exposed on serosal surface; si, infiltration beyond the serosa to other tissues; ss, subserosal tissue; Tub1, well differentiated tubular adenocarcinoma; Tub2, moderately differentiated tubular adenocarcinoma.

Supplementary Table 5. Incidence of intraepithelial spread beyond borders in cystic duct cancers (CyDCs), gallbladder cancers (GBCs), and ampulla of Vater cancers (AVCs).

| CyDC | total | conventional CyDC | ICPN |
| --- | --- | --- | --- |
| IES on gallbladder (+) | 13 | 8 | 5 |
| IES on EHBD (+) | 8 | 4 | 4 |
| total/ */ ** | 20/ 18/ 13 | 15/ 13/ 8 | 5/ 5/ 5 |

*CyDC reached to the border between gallbladder and CyD. **CyDC reached to the junction of CyD.

| GBC | total | conventional GBC | ICPN |
| --- | --- | --- | --- |
| IES on CyD (+) | 14 | 10 | 4 |
| IES on EHBD (+) | 2 | 1 | 0 |
| total/ ¶/ ¶¶ | 172/ 18/ 13 | 120/ 16/ 1 | 52/ 4/ 0 |

¶GBC reached to the border between gallbladder and CyD. ¶¶GBC reached to the junction of CyD.

| AVC | total | conventional AVC | IAPN |
| --- | --- | --- | --- |
| IES on EHBD (+) | 23 | 11 | 12 |
| IES on MPD (+) | 17 | 7 | 10 |
| intraepithelial spread on duodenum (+) | 17 | 1 | 16 |
| total | 102 | 50 | 52 |

AVC, ampulla of Vater cancer; EHBD, extrahepatic bile duct; CyD, cystic duct; CyDC, cystic duct cancer; GBC, gallbladder cancer; IAPN, intraampullary papillary tubular neoplasm; ICPN, intracholecystic papillary neoplasm; IES, intraepithelial spread; MPD, main pancreatic duct.

Supplementary Table 6. Intraepithelial spread beyond ampullary area in ampullary cancers.

|  | Conv. AVC  n= 50 | Conv. AVC  n= 50 | IAPN  n= 52 | IAPN  n= 52 |
| --- | --- | --- | --- | --- |
| IES, mm | IES on EHBD | IES on MPD | IES on EHBD | IES on MPD |
| 0 | 39 (78.0) | 43 (86.0) | 40 (76.9) | 42 (80.8) |
| 0 <, < 10 | 6 (12.0) | 5 (10.0) | 7 (13.5) | 7 (13.5) |
| 10 ≤, < 20 | 3 (6.0) | 2 (4.0) | 3 (5.8) | 2 (3.8) |
| 20 ≤, < 30 | 1 (2.0) | 0 | 1 (1.9) | 1 (1.9) |
| 30 ≤, < 40 | 0 | 0 | 0 | 0 |
| 40 ≤, < 50 | 0 | 0 | 1 (1.9) | 0 |
| 50 ≤, < 60 | 0 | 0 | 0 | 0 |
| 60 ≤ | 1 (2.0) | 0 | 0 | 0 |

Values given are the number of patients (percentage) unless otherwise indicated.

AVC, ampulla of Vater cancer; EHBD, extrahepatic bile duct; IAPN, intraampullary papillary tubular neoplasm; IES, intraepithelial spread; MPD, main pancreatic duct.

**Supplementary Figure legends**

Supplementary Figure 1. Selection of extrahepatic cholangiocarcinoma (eCCA) cases. Bph, Bs, Bm, and Bi indicate (peri)hilar bile duct, superior portion of extrahepatic bile duct (EHBD), middle portion of EHBD, and inferior portion of EHBD. IPNB indicates intraductal papillary neoplasm of the bile duct.

Supplementary Figure 2. Kaplan–Meier survival curves and survival rates of extrahepatic cholangiocarcinomas (eCCAs). Left and right panels show recurrence-free survival and overall survival, respectively. Kaplan–Meier curves of total invasive eCCAs (blue), Bs eCCAs (orange), Bm eCCAs (green), Bi eCCAs (red), invasive IPNBs (black), and non-invasive IPNBs (pink) are compared. Differences are examined by a log-rank test. *, *P*< 0.05; **, *P*< 0.01; ***, *P*< 0.001.

Supplementary Figure 3. Kaplan–Meier survival curves and survival rates of invasive extrahepatic cholangiocarcinomas (eCCAs) with or without intraepithelial spread (IES). Upper and lower panels show recurrence-free survival and overall survival, respectively. Kaplan–Meier curves of each location of conventional eCCAs or invasive IPNBs with IES (red) and without IES (blue) are compared. Differences are examined by a log-rank test. *, *P*< 0.05; **, *P*< 0.01; ***, *P*< 0.001.

Supplementary Figure 4. Features of invasive extrahepatic cholangiocarcinoma in which cancer cells occupied the junction of cystic duct. Gray color indicates cancer area with stromal invasion of cancer cells, and red color indicates intraepithelial spread of cancer cells without stromal invasion.

Supplementary Figure 5. Kaplan–Meier survival curves and survival rates. Left and right panels show recurrence-free survival (RFS) and overall survival (OS), respectively. Kaplan–Meier curves of group A (red), group B (green), and group C (blue) are compared in total invasive extrahepatic cholangiocarcinomas (eCCAs) (A), in invasive conventional eCCAs (B), in perihilar eCCAs (C), and in distal eCCAs (D). Differences are examined by a log-rank test. *, *P*< 0.05; **, *P*< 0.01; ***, *P*< 0.001.
