# Supplementary figures and images for: Novel insights into the intraepithelial spread of extrahepatic cholangiocarcinoma: clinicopathological study of 382 cases on extrahepatic cholangiocarcinoma
Source: Front Oncol. 2023 Aug 17;13:1216097. doi: 10.3389/fonc.2023.1216097 (PMC10470634; doi:10.3389/fonc.2023.1216097)

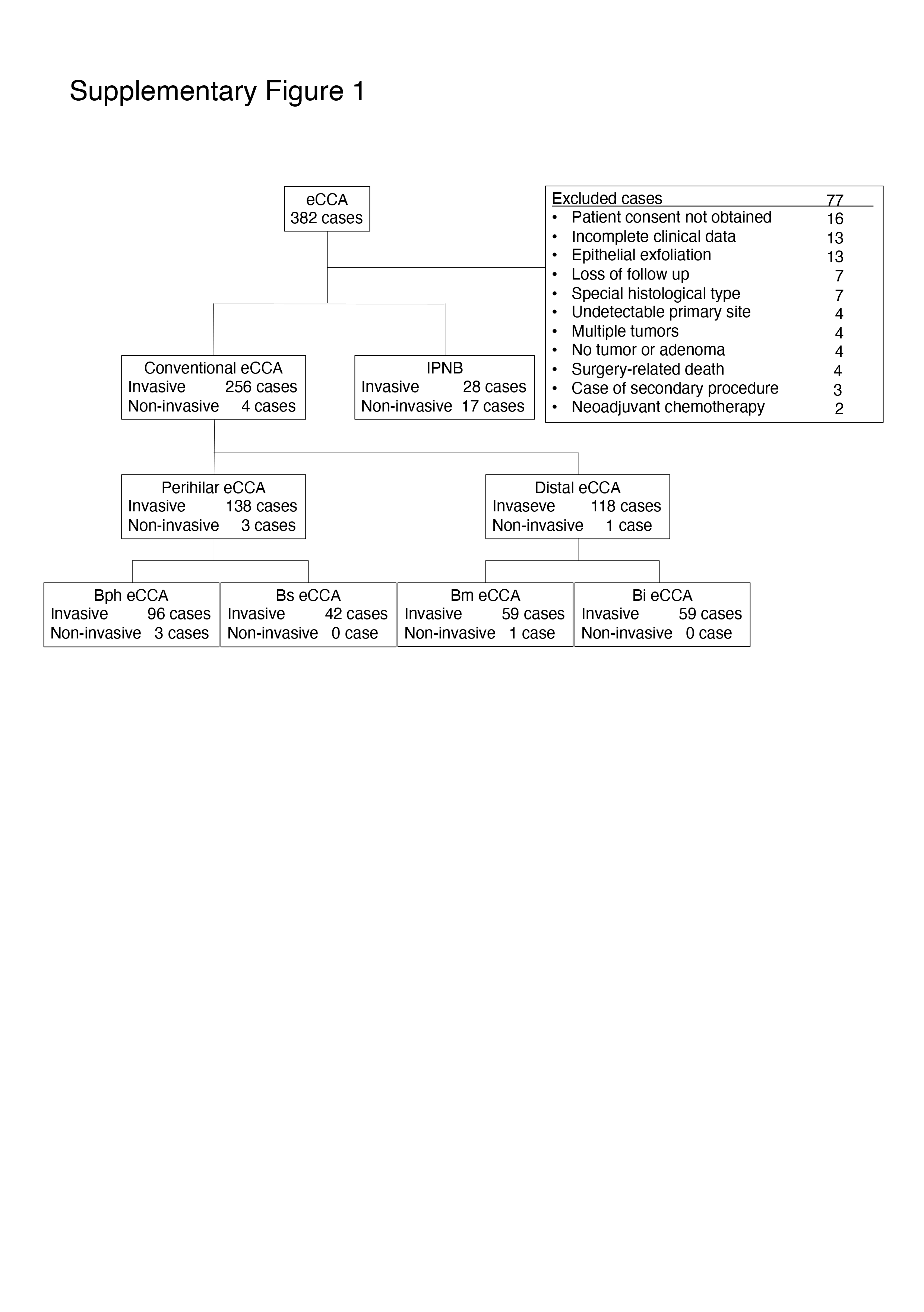

Supplement: Supplementary file 2 [file Image_1.tif]

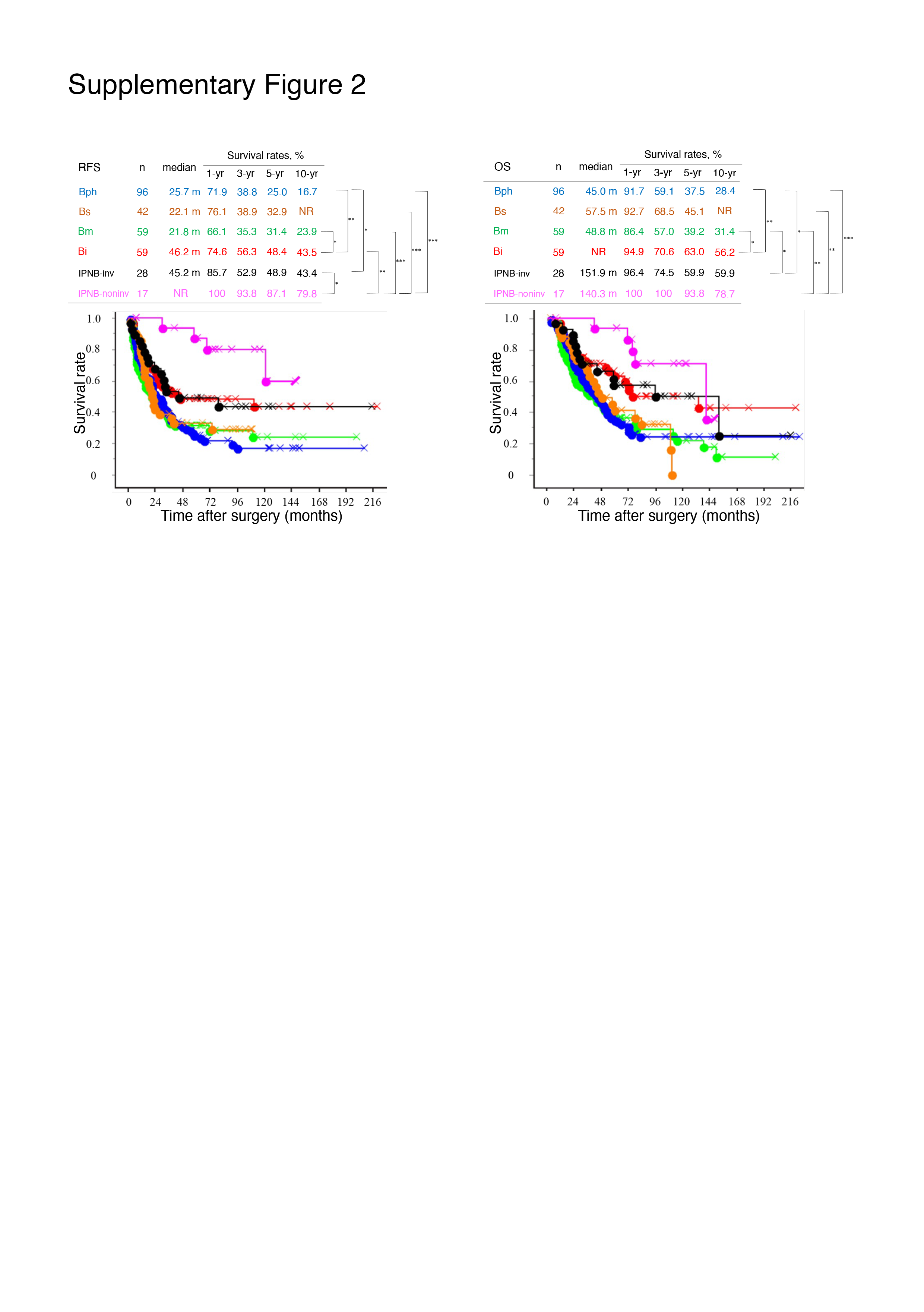

Supplement: Supplementary file 3 [file Image_2.tif]

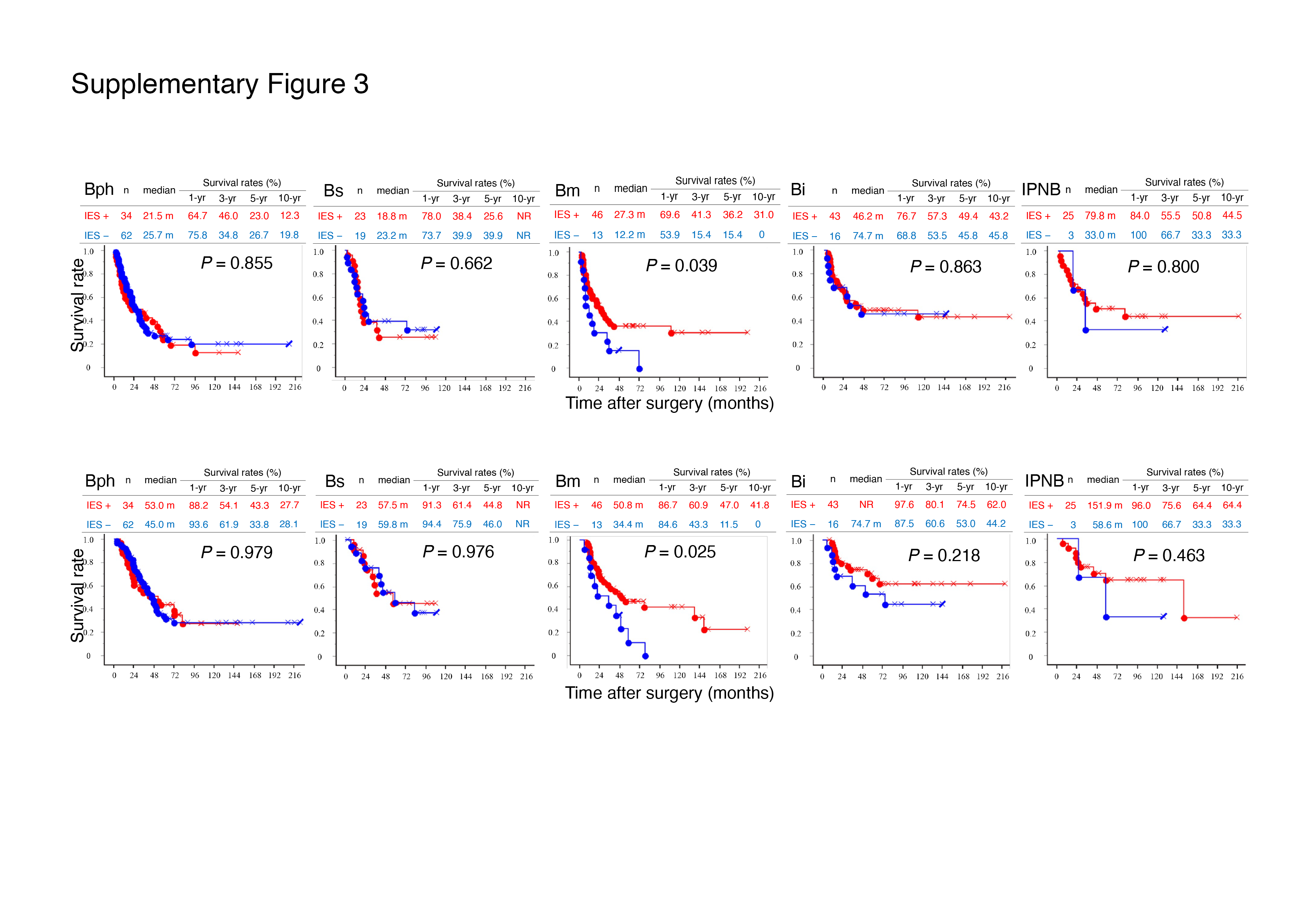

Supplement: Supplementary file 4 [file Image_3.tif]

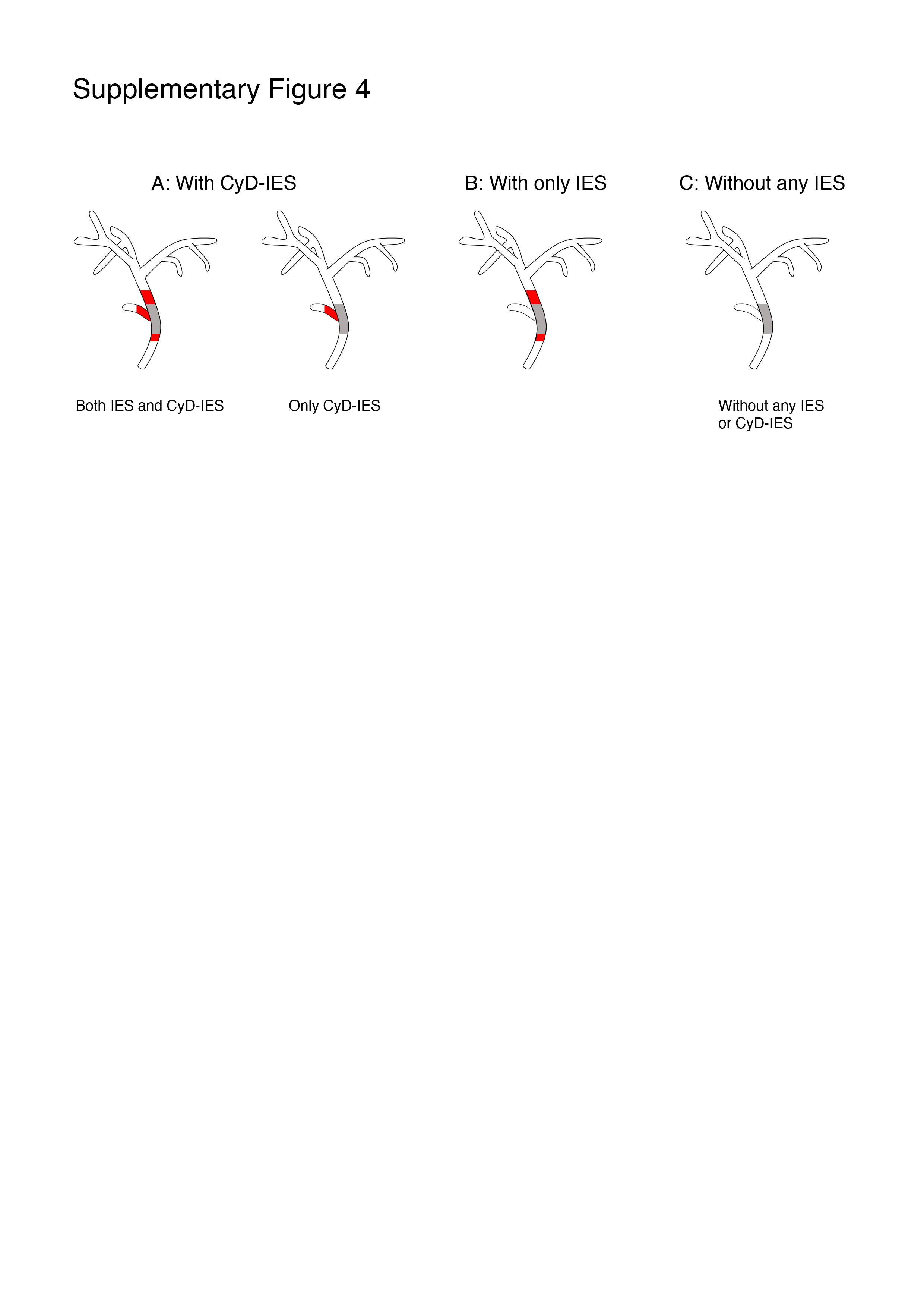

Supplement: Supplementary file 5 [file Image_4.tif]

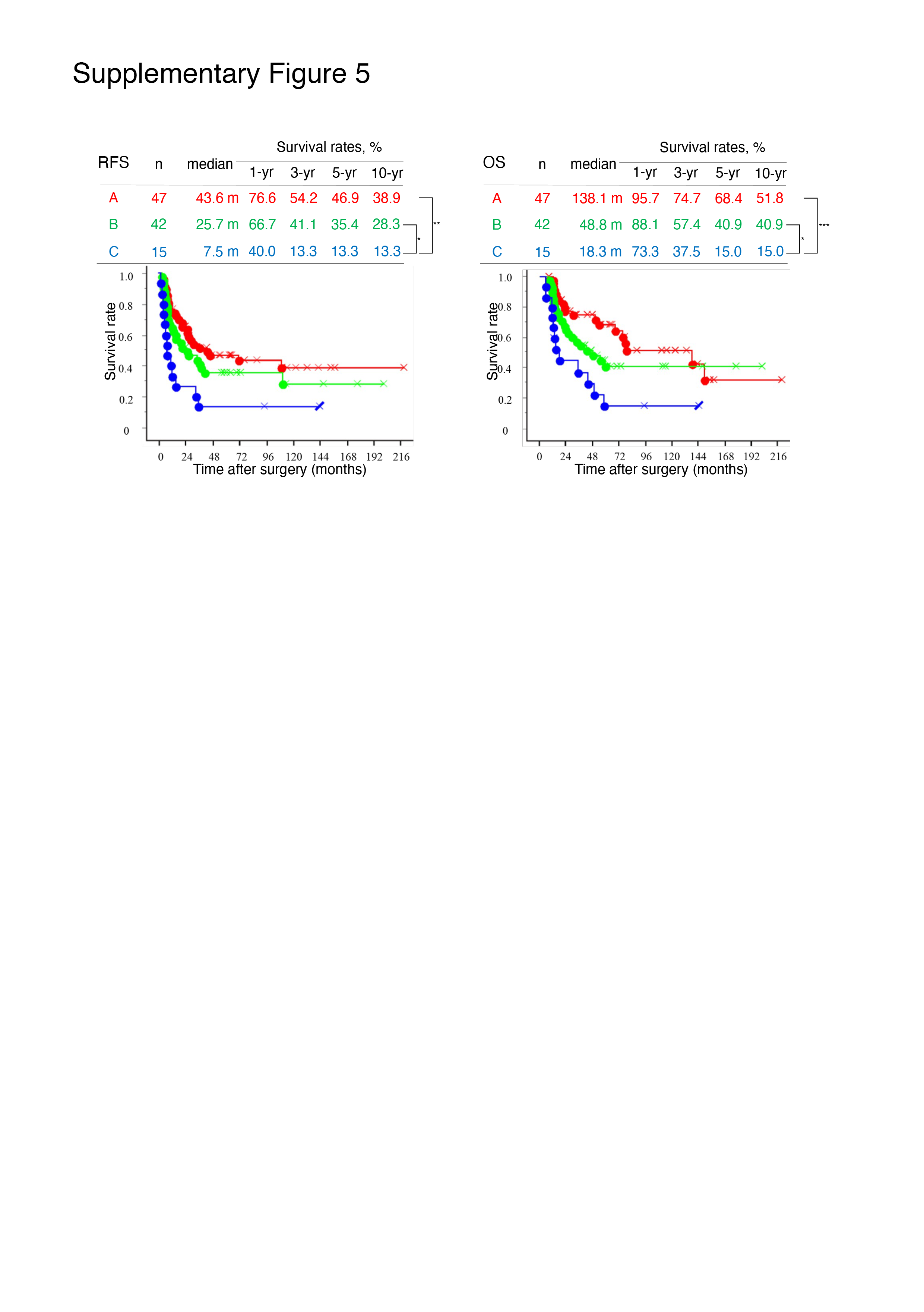

Supplement: Supplementary file 6 [file Image_5.tif]
